# Unveiling New Insights: Reinterpreting *DES* Mutation, p.Arg383His, Through a Study of an Iranian Family With Isolated Hypertrophic Cardiomyopathy, Implication for Phenotype–Genotype Correlation Analysis

**DOI:** 10.1002/ccr3.73051

**Published:** 2026-07-28

**Authors:** Saeideh Kavousi, Farzad Kamali, Bahareh Rabbani, Mehrdad Behmanesh, Nejat Mahdieh, Mehrdad Noruzinia

**Affiliations:** ^1^ Department of Medical Genetics, Faculty of Medical Sciences Tarbiat Modares University Tehran Iran; ^2^ Rajaie Cardiovascular Medical and Research Center Iran University of Medical Sciences Tehran Iran; ^3^ Growth and Development Research Tehran University of Medical Sciences Tehran Iran; ^4^ Department of Genetics, Faculty of Biological Sciences Tarbiat Modares University Tehran Iran; ^5^ Cardiogenetic Research Center, Rajaie Cardiovascular Medical and Research Center Iran University of Medical Sciences Tehran Iran

**Keywords:** cardiomyopathy, DCM, DES, HCM, phenotypic variability

## Abstract

Desmin, a crucial intermediate filament in muscle cells, maintains structural integrity in cardiac muscle and provides stability to striated muscle cells. Mutations in the *DES* gene lead to desminopathies, causing diverse cardiac and skeletal myopathies. We examine an Iranian family with a highly penetrant p.Arg383His variant in the *DES* gene, resulting in severe hypertrophic cardiomyopathy (HCM) without skeletal phenotypes. Moreover, we discuss all reported disease‐causing missense variants, examining their clinical manifestations across different domains. We assessed demographic data, clinical features, and genetic analysis in members of this Iranian family. Whole genome sequencing (WGS), in silico structural and functional predictions, was also used to investigate genetic entities. Also, a mini‐review was performed across various databases to identify all disease‐causing missense variants within the *DES* gene. WGS identified a p.Arg383His variant in the *DES* gene in the Iranian family. Analyzing 119 disease‐causing missense variants in desmin revealed limited correlation between variant location and phenotypes. A significant prevalence (36.9%) of conduction diseases was linked to variants in various domains. Heart failure appeared enriched among variants located in coil2B, while syncope was more frequently reported in variants affecting coil2B and the tail domains; however, these observations are exploratory and literature‐derived. Different domains showed varying associations with specific clinical outcomes, such as spine ankylosis in the tail domain and dysphonia in the desmin head domain. The present study reports an Iranian family exhibiting severe HCM due to a likely pathogenic *DES* gene variant, lacking skeletal myopathy phenotypes. Also, examination of all missense variants highlighted clinical heterogeneity and complex inheritance patterns among individuals carrying different variants in the *DES* gene. In this context, genetic analysis is a valuable diagnostic tool for effectively managing affected patients, identifying carriers, and facilitating future family planning decisions.

## Key Clinical Message

The identification of the likely pathogenic p.Arg383His variant in *DES* supports its probable role in isolated hypertrophic cardiomyopathy in this family. Comprehensive genetic analysis, particularly whole‐genome sequencing, proves indispensable for accurate diagnosis, clinical management, carrier detection, and informed reproductive decision‐making in hereditary cardiac conditions characterized by phenotypic heterogeneity and complex inheritance patterns.

## 1 Introduction

Mutations in *DES* encompass cardiomyopathies such as dilated cardiomyopathy (DCM) [1], hypertrophic cardiomyopathy (HCM) [2], restrictive cardiomyopathy (RCM) [3], left ventricular noncompaction cardiomyopathy (LVNC) [4], and arrhythmogenic right ventricular cardiomyopathy (ARVC) [5]. Less than 1% of reported patients present with cardiac conduction disease, characterized by ventricular arrhythmia and/or atrioventricular block, which can result in sudden death [6]. Nearly 50% of carriers of *DES* gene variants experience cardiomyopathy. In terms of disease progression, patients with HCM tend to fare worse than those with DCM, as evidenced by a younger age at diagnosis, a greater frequency of heart transplantation, and earlier mortality [7].

Desmin, a protein encoded by the *DES* gene, plays a crucial role in orchestrating the intracytoplasmic filamentous network, establishing connections between myofibrils and the sarcolemma as well as the structural arrangement of desmosomes and the nuclear envelope within cardiac, skeletal, and smooth muscle tissues [8]. To date, 184 disease‐causing genetic variants have been described in this gene (https://www.hgmd.cf.ac.uk/ac/index.php); approximately 65% of these variants are missense, and the remaining are nonsense, small indels, splicing, large deletions, and complex rearrangements. These variations lead to the accumulation of insoluble filamentous material in the cytoplasm of muscle cells, disrupting the crosstalk between the extracellular and nuclear matrix and impeding the filament assembly process [9]. Consequently, diverse phenotypes, collectively referred to as desminopathies, can arise [10]. Desminopathies exhibit different combinations of heterogeneous phenotypes, including skeletal myopathies such as myofibrillar myopathy (MFM) [11], neurogenic scapuloperoneal syndrome (Kaeser type) [12], and limb‐girdle muscular dystrophy (LGMD2R) [13].

Nonetheless, the specific molecular mechanisms by which *DES* variants contribute to the development of these diseases are unclear. Notably, not all pathogenic *DES* variants lead to aggregate formation, suggesting the potential involvement of both loss‐of‐function and toxic gain‐of‐function mechanisms. The inheritance of desminopathies follows an autosomal dominant pattern, although patients with autosomal recessive inheritance have rarely been reported [14].

Even if there are currently no definitive genotype–phenotype correlations established for these phenotypes, the severity of skeletal and myocardial involvement, age of onset, and disease progression can vary depending on the specific variant locations and inheritance. Diseases associated with biallelic desmin variants often exhibit a more severe phenotype. The occurrence of cardiomyopathy preceding myopathic features, although rare, introduces additional challenges to clinical diagnosis [15].

We herein describe phenotypic variabilities due to missense variants in this gene. The different phenotypes of all disease‐causing missense variants and their frequencies were analyzed to evaluate the distribution of missense variations within the desmin protein domains and their associated phenotypes. In addition, an Iranian family affected by severe HCM and cardiac conduction complications was reported. Notably, the affected individuals did not exhibit any concurrent skeletal myopathy and needed implantable cardioverter‐defibrillator (ICD) placement for management. To perform a comprehensive genetic analysis, we used WGS as a sensitive method to identify rare and common genetic variants in HCM patients [16].

## 2 Case History/Examination

### 2.1 Family Presentation

A 14‐year‐old proband (IV‐1) was a male patient referred to Rajaie Cardiovascular Medical and Research Hospital due to hypertension (140/90 mmHg) and dyspnea. During a detailed nursing assessment, there was no recorded syncope, seizures, or cyanosis in the patient's medical history, and no physical lesions were identified during the examination. The patient exhibited normal motor developmental milestones.

A comprehensive analysis of blood count; alanine transaminase, aspartate transaminase, alkaline phosphatase, and serum protein levels; serum creatinine; and serum electrolyte levels revealed results within the normal range. Her creatine kinase (CK) level was normal at 83 U/L (normal range 25–300 U/L). However, the pro‐B‐type natriuretic peptide (pro‐BNP) level was significantly elevated at 4129 pg/mL, exceeding the normal cutoff (125 pg/mL) and indicating a risk of heart failure. General urine analysis revealed a trace amount of blood and 4–5 red blood cells per high‐power field in the patient's urine.

**FIGURE 1:**
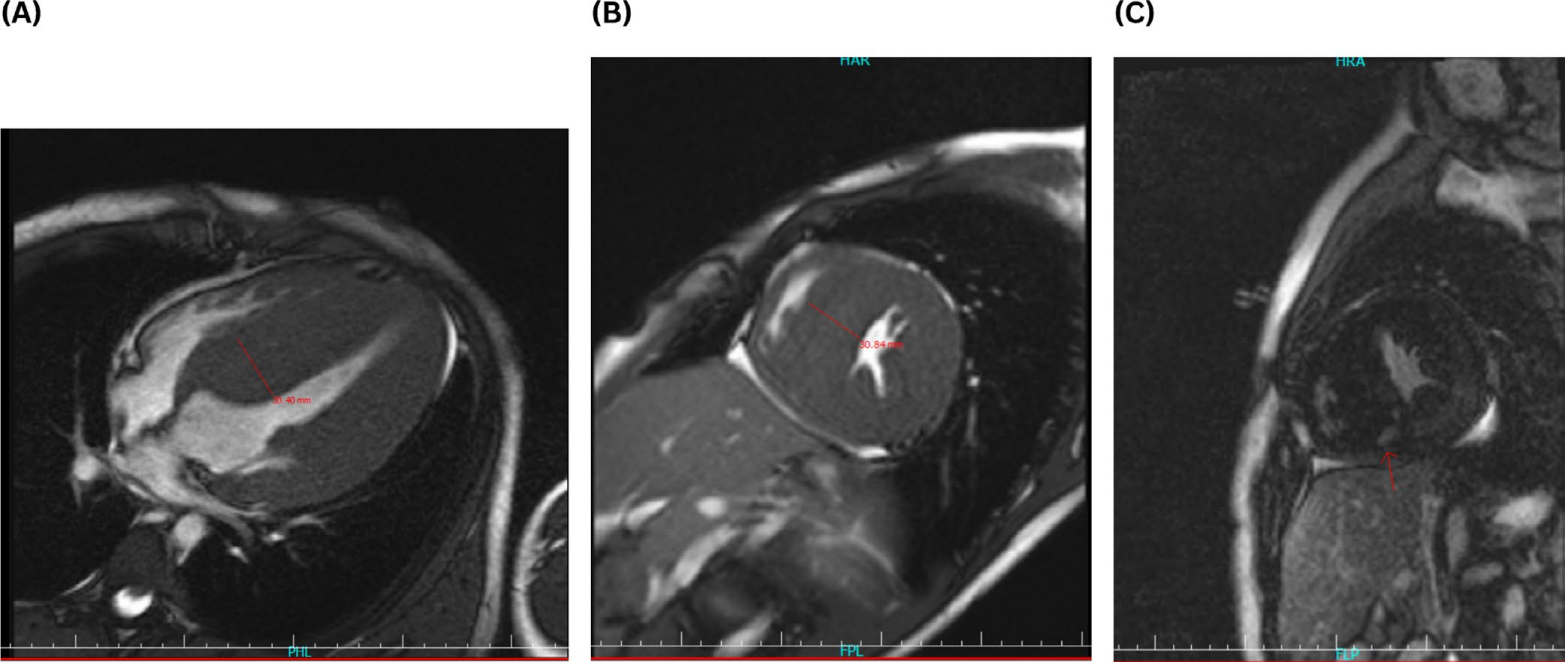
The clinical information of the patient. (A) Steady‐state free precession (SSFP) in four chambers and (B) short‐axis views showing typical asymmetrical septal hypertrophy with a maximum wall thickness of 30 mm. (C) Late gadolinium enhancement (LGE) revealed localized patchy fibrosis in the inferoseptal region of the left ventricle.

Although there was no family history of neuromuscular disorders, the presence of cardiac complications in multiple family members strongly suggested the presence of hereditary cardiac diseases. Pediatric transthoracic echocardiography revealed severe hypertrophy of the interventricular septum (IVS > 3.2 cm) and a left ventricle‐free wall thickness of 4 cm. Mild to moderate mitral regurgitation, mild tricuspid regurgitation, and a left ventricular ejection fraction (LVEF) ranging from 55% were observed. Additionally, left ventricular outflow tract (LVOT) gradients of 20 mmHg, an aortic valve area (AO VTI) of 20 cm, and mild to moderate pulmonary insufficiency (PI) with a pressure gradient of 20 mmHg were detected. No evidence of coarctation of the aorta (COA) was noted. Based on the echocardiogram report, cardiovascular magnetic resonance (CMR) results, and the patient's clinical indications, HCM was diagnosed, and treatment with beta‐blockers and angiotensin‐converting enzyme (ACE) inhibitors was initiated (Figure 1).

**FIGURE 2:**
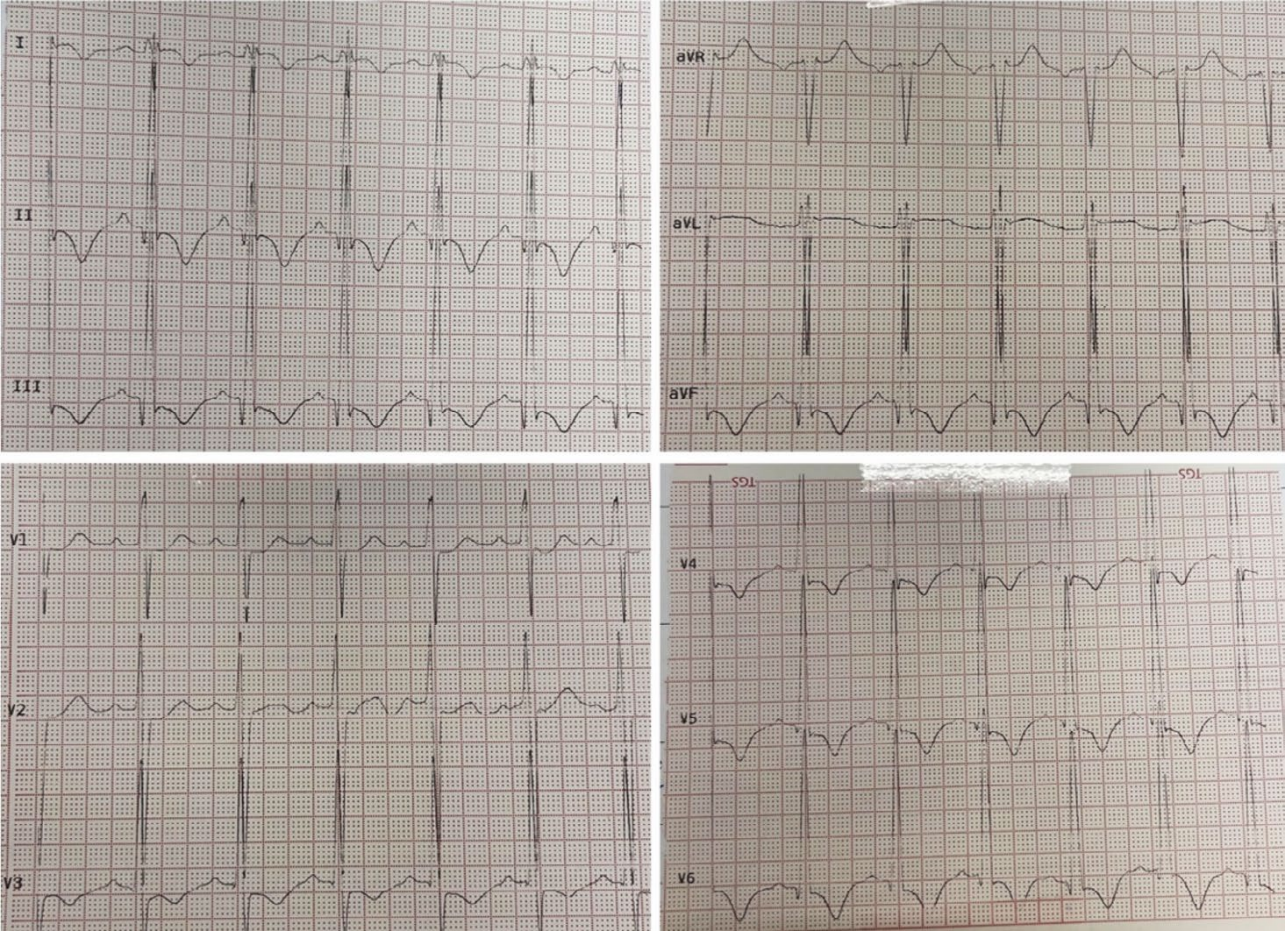
Twelve‐lead ECG report. ECG revealed a normal sinus rhythm with right axis deviation, narrow Q waves in the inferior leads, an RS pattern in V1–2, voltage criteria for left ventricular hypertrophy, inverted T waves in the inferior and lateral leads, and a prolonged QT interval.

A twelve‐lead electrocardiogram (ECG) was used to record a normal sinus rhythm with right axis deviation, narrow Q waves in the inferior leads, an RS pattern in V1‐2, voltage criteria for left ventricular hypertrophy (LVH), inverted T waves in the inferior and lateral leads, and a prolonged QT interval (Figure 2).

CMR was performed to assess HCM in the left ventricle (LV). The evaluation of cardiac morphology revealed normal sizes for both the left and right atria, with no evidence of thrombus formation. The LVs were normal in size but exhibited an increased total LV mass. The systolic function of the patients remained preserved, with an LVEF of 51%. Additionally, the assessment revealed asymmetrical LVH characterized by thickening in the mid‐anteroseptal wall, reaching a maximal septal thickness of 30 mm, consistent with asymmetric septal hypertrophy (ASH). On the other hand, the right ventricle (RV) was normal in size, without evidence of right ventricle hypertrophy (RVH), and preserved systolic function, with a right ventricular ejection fraction (RVEF) of 53%. Velocity flow mapping assessment of the cardiac valves revealed normal functioning of the aortic, tricuspid, and pulmonic valves, along with mild regurgitation in the mitral valve. An abnormality known as systolic anterior motion (SAM) of the anterior mitral valve leaflet (AMVL) was observed, resulting in mitral regurgitation, turbulence, and obstruction in the LVOT.

Furthermore, thoracic cage magnetic resonance angiography (MRA) with myocardial assessment showed no signs of dissection, aneurysm, or significant stenosis. The supra‐aortic vessels displayed an unremarkable course and diameter, while the pulmonary arteries appeared normal in caliber and appearance. A late gadolinium enhancement study revealed a specific pattern of localized patchy mid‐myocardial fibrosis at the RV septal junctions, consistent with HCM. The assessment of scar tissue, calculated using a 5‐point standard deviation threshold, revealed a difference of 8.27%, indicating the extent of fibrotic remodeling in the myocardium. Over subsequent years, with the implementation of standard treatments for the management of HCM, the patient's condition has deteriorated over time.

A tissue Doppler echocardiography report revealed several notable findings. These included severe LVH characterized by ASH, hypertrophic papillary muscles, and severe SAM of the mitral valve. Moreover, mid‐cavity obliteration was observed, resulting from significant hypertrophy of the left ventricular wall and papillary muscles and leading to marked dynamic obstruction. The peak gradient was 67 mmHg across the mid‐left ventricular cavity and LVOT. Furthermore, significant RVH was observed, with the anterior right ventricular wall measuring 1.2 cm. Systolic turbulence was noted in the right ventricular outflow tract (RVOT) due to hypertrophy of the anterior right ventricular wall, although no obstruction was present (peak gradient: 15 mmHg).

Based on the cardiologist's diagnosis, a dual‐chamber ICD was implanted to enhance the management of obstructive hypertrophic cardiomyopathy (HOCM). The implantation procedure was performed after the appropriate preimplantation screening.

Patient IV‐1's mother (III‐8), a 45‐year‐old woman, claimed to have mild heart muscle disease consistent with HCM during the genetic counseling session. We did not have access to her clinical documentation, but we had her blood sample available for genetic analysis. During the genetic counseling session, the family provided information indicating the presence of suspected cardiac disorders in the siblings of the patient's mother (III‐10, III‐11, III‐14).

Among the family members, individuals III‐10 and III‐14 were diagnosed with hypertension. It is worth mentioning that the individual (III‐11) and proband's cousin (IV‐4) also underwent ICD implantation, highlighting the severity of their cardiac condition. Moreover, the paternal grandfather of the proband (II‐4) underwent open heart surgery, and individual III‐1 experienced heart failure accompanied by a ventricular septal defect, further emphasizing the complex cardiac issues within the family lineage.

## 3 Differential Diagnosis, Investigations

### 3.1 Genetic Analyses

#### 3.1.1 Whole‐Genome Sequencing

The standard salting‐out technique was followed to extract DNA samples from the family members [17]. Whole‐genome sequencing was carried out on proband DNA to achieve at least 30‐fold coverage, with ≥ 95% of bases sequenced to at least 8 × coverage, using an Illumina NovaSeq 6000 sequencer (Illumina, CA, USA) via Dante Labs Inc. (L'Aquila, Italy). The DRAGEN Bio‐IT Platform was used to generate raw data files, which included FASTQ R1, FASTQ R2, the binary alignment map (BAM), and the variant call format (VCF) of single nucleotide polymorphisms (SNPs), insertion–deletion variants (indels), and copy number variations (CNVs). Variant quality score recalibration (VQSR) was performed using the Genome Analysis Toolkit (GATK) variant recalibration to filter possible artifacts in the calls. SNPs and indels were filtered with VQSR sensitivity thresholds of 99.5% and 99.0%, respectively. Genotype quality (GQ) (≥ 20 ×) and read depth (DP) (≥ 10 ×) were additionally used to filter out SNPs with erroneous variant calls, while indels were also needed to pass GQ (≥ 20 ×) and DP (≥ 10 ×). The VCF files were annotated using the Ensembl Variant Effect Predictor (VEP) command line tool (https://github.com/Ensembl/ensembl‐vep) [18]. Significant CNVs were identified using CNVnator via a combination of statistical and machine learning methods to detect CNVs based on the read depth of each smaller region of the genome.

#### 3.1.2 Pathogenicity Interpretation

In silico prediction scores were obtained through the following technique to study the pathogenicity of different variants: MutationTaster (http://www.mutationtaster.org/) [19].

The mutation assessor (http://mutationassessor.org/) [20] and combined annotation‐dependent depletion (CADD) (https://cadd.gs.washington. edu/) [21], deleterious annotation of genetic variants using neural networks (DANN) (https://cbcl.ics.uci.edu/public_data/DANN/.) [22], EIGEN (http://www.columbia.edu/~ii2135/eigen.html) [23], MutPred2 (http://mutpred.mutdb.org/) [24], Missense Variant Pathogenicity prediction (MVP) (https://github.com/ShenLab/missense) [25], Polymorphism Phenotyping v2 (PolyPhen2) (http://genetics.bwh.harvard.edu/pph2/) [26], Sorting Intolerant From Tolerant (SIFT) (https://sift.bii.a‐star.edu.sg/) [27], Functional Analysis through Hidden Markov Models (FATHMM‐MKL) (http://fathmm.biocompute.org.uk/fathmmMKL.htm) [28], Likelihood Ratio Test (LRT) (http://genetics.wustl.edu/jflab/lrt_query.html) [29], Mendelian Clinically Applicable Pathogenicity (M‐CAP) (http://bejerano.stanford.edu/MCAP/) [30], CardioBoost (https://www.cardiodb.org/cardioboost/) [31], MetaRNN (http://www.liulab.science/metarnn.html) [32], Rare Exome Variant Ensemble Learner (REVEL) (https://sites.google.com/site/revelgenomics) [33], BayesDel (https://fengbj‐laboratory.org/BayesDel/BayesDel.html) [34] and GenoCanyon (https://zhaocenter.org/GenoCanyon_Index.html) [35]. Genomic conservation scores were obtained from the following programs: Phylogenetic *p‐*value from the Phylogenetic Analysis with Space/Time models (PHAST) package (http://compgen.cshl.edu/phast/) for multiple alignments of 99 vertebrate genomes to the human genome (phyloP100way_vertebrate) [36], Genomic Evolutionary Rate Profiling (GERP) (http://mendel.stanford.edu/SidowLab/downloads/gerp/) [37] and phastCons (http://compgen.cshl.edu/phast/) [38].

The population frequency was further evaluated through comparison with the variants reported in the Genome Aggregation Database (gnomAD) (https://gnomad.broadinstitute.org/) and Iranome (http://www.iranome.ir/). ClinVar (https://www.ncbi.nlm.nih.gov/clinvar/) and the Human Gene Mutation Database (HGMD) (http://www.hgmd.cf.ac.uk/ac/index.php), were used to identify previously reported variants. Rare protein‐coding SNPs, indels, and CNVs were evaluated for pathogenicity utilizing the American College of Medical Genetics (ACMG) and Association for Molecular Pathology (AMP) standards [39, 40].

### 3.2 Family Screening and Sanger Sequencing

Validation of the p.Arg383 variant involved the creation of specific primers using Primer3 (https://bioinfo.ut.ee/primer3‐4.1.0/). These primers, designated DES‐F (5′‐gtggctaccaggacaacattg‐3′) and DES‐R (5′‐ggtaatcagtaatctcgagcc‐3′), were employed to amplify the target sequences. The employed Sanger sequencing protocol was adapted from our prior publication [41].

The sequencing results were analyzed using 4Peaks and subsequently cross‐referenced with the *DES* gene sequence from the NCBI database (NM_001927.4).

### 3.3 In Silico Protein Structure Prediction and Visualization

The DES protein sequence was aligned using UniProtKB/Swiss‐Prot P17661. DES protein domains were determined with the Simple Modular Architecture Research Tool (SMART) (https://smart.embl.de/smart/show_motifs.pl?ID=P17661).

The complete structure of the DES protein (AF‐P13473‐F1‐model‐v4) was obtained from the AlphaFold Protein Structure Database with high confidence [42].

Prediction of protein stability changes for the p.Arg383His variant was performed by the web server tool MUpro‐UCI (http://mupro.proteomics.ics.uci.edu/) [43].

The homology/analogy recognition engine V2.0 (Phyre2) [44] and the DynaMut server [45] were utilized to assess the impact of potential variants on protein structure and function. The three‐dimensional (3D) structure of wild‐type and mutant desmin was visualized by DynaMut. An additional conservational study was conducted via the Consurf server [46].

### 3.4 Data Extraction and *DES* Gene Missense Variant Identification

A comprehensive search was conducted to identify disease‐causing missense variants with pathogenic/likely pathogenic pathogenicity in the *DES* gene. We searched for the following keywords for a literature review: *DES* gene [title/abstract], missense *DES* gene [title/abstract], desminopathy [title/abstract], cardiomyopathy *DES* gene [title/abstract], and skeletal myopathy *DES* gene [title/abstract] through ClinVar (https://www.ncbi.nlm.nih.gov/clinvar/), VarSome (https://varsome.com/), HGMD (https://www.hgmd.cf.ac.uk/ac/index.php), and NCBI PubMed to identify all the published and unpublished variants up to March 2024. In addition, in PubMed we applied the Boolean search strategy (“DES” OR “desmin”) AND (“missense” OR “missense mutation” OR “missense variant”) AND (“cardiomyopathy” OR “myopathy” OR “desminopathy” OR “arrhythmia”) to ensure comprehensive retrieval. The abovementioned databases were investigated, and duplicate records were excluded to avoid errors associated with overrepresentation. When the same variant was reported in multiple databases or publications, it was counted once and clinical information was consolidated.

All variants were named and annotated following the Human Genome Variation Database (HGVS) and canonical *DES* transcript NM_001927.4. The ACMG categorization of each variant was reported based on VarSome. ACMG classifications were cross‐checked with ClinVar submissions and HGMD entries when available. In cases of conflicting interpretations, priority was given to expert‐reviewed ClinVar submissions and peer‐reviewed publications applying ACMG criteria. Variants classified as benign, likely benign, or variants of uncertain significance (VUS) were excluded from the final dataset. In addition, synonymous, nonsense, frameshift, splice‐site, intronic, and structural variants were excluded to restrict the analysis to missense substitutions.

Clinical symptoms associated with each variant were collected from relevant articles and databases to provide comprehensive information. Extracted phenotypic data were grouped into major clinical domains, including cardiomyopathy subtype, conduction disease, arrhythmia, heart failure, sudden cardiac death, skeletal myopathy, respiratory involvement, and dysphonia, to enable comparative genotype–phenotype assessment. Variants reported exclusively in obligate digenic or trigenic contexts were not included unless independent evidence supported a primary pathogenic contribution of the DES variant; when additional variants were present, this was documented.

The table includes the dbSNP database rs# ID, ClinVar variation ID, ClinVar submitted interpretations, and references for each variant. For more detailed information, please refer to the table provided in Table S1.

As this review is based on published literature and database submissions, the findings may be influenced by publication bias and variability in clinical characterization, and therefore genotype–phenotype observations should be interpreted with caution.

## 4 Conclusion and Results

### 4.1 Conclusion

The present study reports an Iranian family displaying severe isolated HCM due to a newly interpreted likely pathogenic p.Arg383His variant in the *DES* gene. The examination of all missense variants has revealed clinical heterogeneity and complex inheritance patterns among carriers of *DES* gene mutations. In this context, our findings underscore the significance of genetic testing, WGS in our study, as a valuable diagnostic tool for efficiently managing affected patients, identifying carriers, and facilitating informed family planning decisions in hereditary cardiac diseases.

### 4.2 Results

#### 4.2.1 Variant Validation and Familial Segregation

Segregation analysis was limited to the affected proband and his affected mother. Other relatives were either unavailable for testing or lacked comprehensive clinical documentation. Although no phenocopies were identified among tested individuals, the restricted number of genotyped relatives limits segregation strength and prevents formal penetrance estimation.

##### 4.2.1.1 Patient IV‐1

**FIGURE 3:**
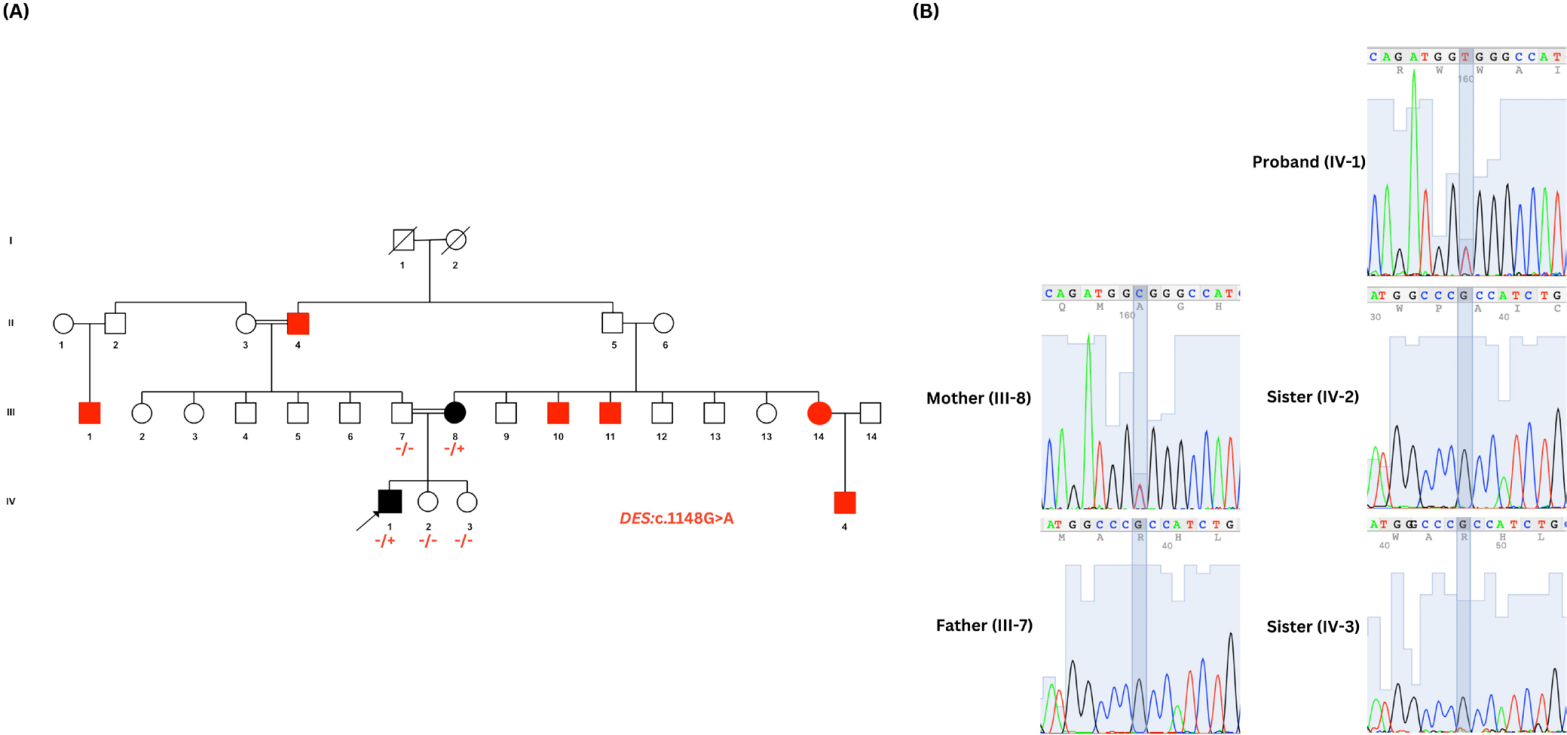
The image illustrates family pedigree and genetic analysis of a DES c.1148G>A variant. (A) Pedigree of the family with the *DES* gene variant. The proband (IV‐1) is indicated by the arrow. The slashed symbols indicate the deceased members. Circles = females; squares = males; black filled shapes = phenotype‐positive subjects with desminopthy; empty shapes = unaffected subjects; red filled shapes = cardiac disorders. (B) The missense variant c.1148G>A in exon 6 of the *DES* gene was found at the heterozygous state in the proband (IV‐1) and his mother (III‐8). The proband's father and sisters carried a normal allele.

The *DES*:c.1148G > A (p.Arg383His) variant was validated through Sanger sequencing, confirming the heterozygous state and autosomal dominant inheritance (Figure 3).

##### 4.2.1.2 Patient III‐8

Familial segregation analysis revealed that the *DES*:c.1148G > A (p.Arg383His) variant originated from the proband's mother. The heterozygous state of the mother, consistent with her phenotypic pattern, confirmed autosomal dominant inheritance. Sanger sequencing verified the absence of the p.Arg383His variant in unaffected family members, including the father and sisters (Figure 3).

### 4.3 Bioinformatic Analysis of p.Arg383His

After conservation analysis utilizing two tools, the PhyloP100way value was 9.5, and the PhastCons100way value was 1.0 (0–1; conserved), demonstrating that p.Arg383His is conserved.

For the c.1148G > A missense variant, computational prediction tools unanimously support a damaging effect on the gene. CADD predicted a PHRED score of 32, which indicated the position of the variant in the top 0.1% of deleterious variants with a base call accuracy of 99.9% (Table 1).

**TABLE 1:** Bioinformatics tools and prediction scores of the *DES*:C.1148G > A (p.Arg383His) variant.

Bioinformatics tools	Prediction	Score
BayesDel addAF	Damaging	0.384
BayesDel noAF	Damaging	0.314
CADD	Pathogenic	32
CardioBoost CM	Damaging	0.97
DANN	Pathogenic	0.999
EIGN	Pathogenic	0.959
FATHMM‐MKL	Damaging	0.991
Frequency in gnomAD	Not found	Not found
GenoCanyon	Deleterious	1
GERP	Uncertain	5.1
gnomAD	Very rare	0.00000398
Iranome	Not found	Not found
LRT	Deleterious	0.00029
M‐CAP	Damaging	0.255
MetaRNN	Damaging	0.905
Mutation Assessor	Medium	3.045
Mutation Taster	Disease causing	0.99
MutPred	Pathogenic	0.732
MVP	Pathogenic	0.974
PhastCons100way	Conserve	1.0
PhyloP100way	Conserve	9.5
Polyphen2 HDIV	N/A	
Polyphen2 HVAR	N/A	
REVEL	Damaging	0.832
SIFT	Damaging	0.007
SNP ID	rs1292042317	—

Abbreviations: CADD, combined annotation‐dependent depletion; DANN, deleterious annotation of genetic variants using neural networks; GERP, genomic evolutionary rate profiling; gnomAD, genome aggregation database; LRT, likelihood ratio test; M‐CAP, Mendelian clinically applicable pathogenicity; MVP, missense variant pathogenicity prediction; PolyPhen‐2, polymorphism phenotyping v2; REVEL, rare exome variant ensemble learner; SIFT, sorting intolerance from tolerant; SNP, single nucleotide polymorphism.

Following the ACMG guidelines, the candidate variant may be categorized as “likely pathogenic” due to its alignment with the PM1, PM2, PP2, and PP3 rules (Table 2).

**TABLE 2:** *DES*: C.1148G > A (p.Arg383His) variant ACMG classification.

ACMG Rule	Strength	Explanation
PM1	Moderate	The nontruncating nonsynonymous variant is situated in a mutational hot spot region and/or critical in the protein's functional domain. Around this variant in exon 6, within the specific range of 220,286,061–220,286,282, a total of ~27 pathogenic or likely pathogenic variants were identified, while no missense benign variants were found. Hot‐spot of length 17 amino‐acids has ~23 missense/in‐frame variants. In the UniProt protein DESM_HUMAN, the ‘Coil 2B’ region of interest exhibited ~126 missense/in‐frame variants. In the UniProt protein DESM_HUMAN, the ‘Interaction with NEB’ region of interest displayed ~155 missense/in‐frame variants.
PM2	Moderate	Extremely low frequency in gnomAD population databases, good gnomAD genomes coverage = 29.5.
PP2	Supporting	Missense variant in a *DES* gene, where benign missense variants are infrequent. It is three times more likely to be pathogenic than a benign variant, suggesting a higher likelihood of causing a disease. Missense mutations are commonly associated with the mechanism of a disease.
PP3	Moderate	computational prediction tools unanimously support a damaging effect on the gene. The MetaRNN score for this variant is 0.905, falling within the range of 0.841 to 0.939, indicating a moderate level of pathogenicity.

*Note:* The information in this table was extracted using the VarSome and Franklin servers.

### 4.4 In silico protein analyses of p.Arg383His

The human DES gene is highly conserved across vertebrates and encodes desmin, a 470 amino acid protein. Structurally, the desmin protein has a central α‐helical rod‐like domain bordered by globular N‐terminal head and C‐terminal tail domains. The isolated rod segment showed 83% α‐helical content and consisted of four segments, termed 1A, 1B, 2A, and 2B, 2A, and 2B, with three linkers. Within the rod domain, two α‐helical rods, which are highly conserved, arrange themselves in parallel form using heptad repeats, forming a left‐handed dimeric coiled coil superhelix [47].

**FIGURE 4:**
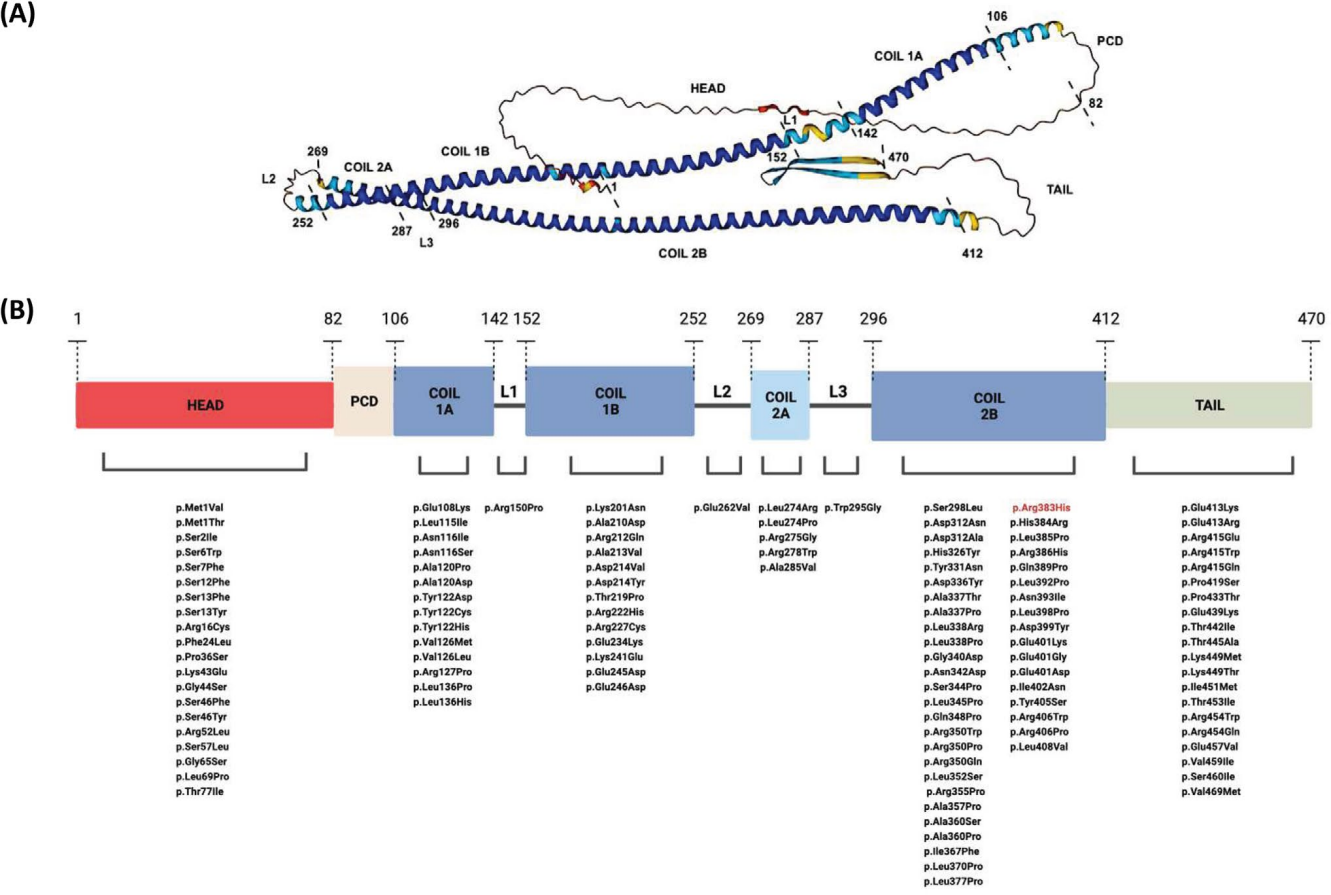
Missense variants distributions in the desmin protein. (A) The position of the domains was determined by the 3D structure of the human desmin protein. (B) Schematic diagram of the desmin protein domains. Missense variants occurring in each of the domains were identified. The location of the Arg383His variant is shown.

The p.Arg383His variant is located in the coil 2B domain, coinciding with positions 296–412 of the desmin protein (Figure 4).

**FIGURE 5:**
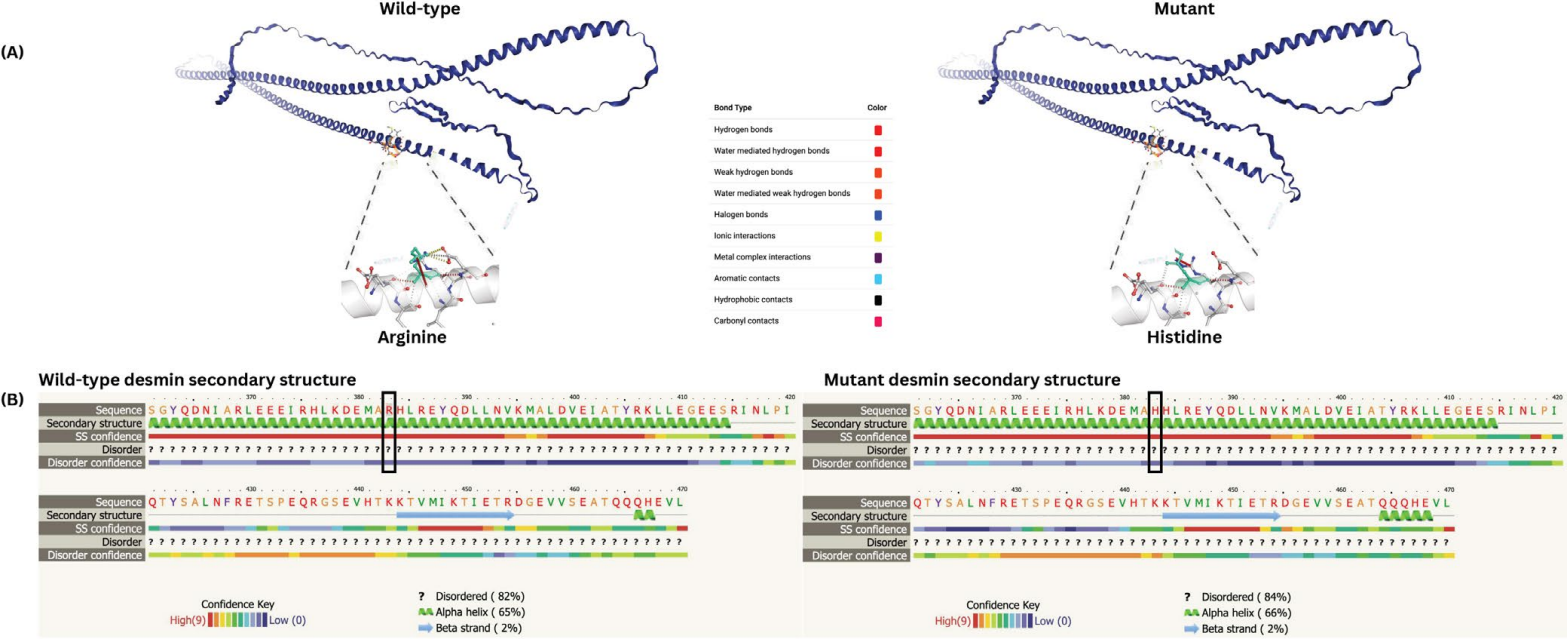
The structure of Desmin protein. (A) 3D structures of wild‐type (left) and mutated (right) DES were generated with the DynaMut server. Wild‐type arginine and mutant histidine are colored light green and are also represented as sticks alongside the surrounding residues, which are involved in any type of interaction. No significant change was observed in the target residue at position 383 or in the surrounding region between the wild‐type and mutant desmin strains. (B) Secondary structure model analysis of the desmin protein using Phyre2. The left column indicates the protein sequence, secondary structure, and disorder for each line of the wild‐type desmin protein (left) and the mutated (p.Arg383His) protein (right). The confidence value score for each item is determined by the confidence key color palette. The desmin mutant exhibited an increase in the percentage of α‐helices, specifically the N‐terminal α‐helix, from 65% to 66%.

The desmin coil 2B domain was modeled using the Phyre2 web portal based on the c1gk4A template (a fragment of human vimentin coil 2B), which resulted in a 77% identity match and a confidence level of 98.7%. The PDB entry 1gk4A represents the local structural environment surrounding the Arg383 residue. Analysis of the secondary structure revealed that wild‐type desmin is predicted to have approximately 65% α‐helices. However, the percentage of α‐helices increased to 66% in the desmin mutant, as shown in Figure 5, especially in the N‐terminal α‐helix. The stability of the α‐helix at amino acid 383 was predicted to be low, with minimal score alterations.

According to the Consurf server, both the wild‐type and mutant alleles exhibited an above‐average conservation score. Additionally, the variant does not alter the exposed state of the amino acids.

3D structure models of both the wild‐type and the mutated form of desmin were generated using Dynamut. Prediction of interatomic interactions revealed only minor changes in ionic interactions and hydrogen bonds, with no significant alterations observed in the desired or surrounding residues between the wild‐type and mutant strains. Analysis of atomic fluctuations, which indicate the extent of atom motion, demonstrated minimal fluctuations at position 383 in both the wild‐type and mutant desmin proteins (Figure 5).

The MUpro‐UCI web server utilizes 3D structure or protein sequence information and relies on a support vector machine (SVM) artificial intelligence approach to predict alterations in protein stability. The prediction model employs protein sequence information to predict the magnitude of energy change. The p.Arg383His variant was associated with a reduction in protein stability. The stability predictor utilizes the ΔΔG value, the difference between the Gibbs free energy (ΔG) of the new protein and the wild type in units of Kcal/mol. The predicted ΔΔG value of the variant was −1.3025 Kcal/mol. Score classification was based on ΔΔG < 0, indicating a decrease in stability, and ΔΔG > 0, indicating an increase in stability.

Analysis of the DES gene revealed that the missense variant p.Arg383His is more likely to cause a functional change than a structural change.

### 4.5 *DES* Gene: Missense Variants Distributions

At the time of this analysis, the total number of classified variants in the *DES* gene was 1125, as documented in both published and unpublished databases. Approximately 65% of these variants are missense variants. We investigated the distribution of missense variants, differentiating those with pathogenic or likely pathogenic variants from other missense variants reported in articles as variants with phenotypes across the various domains of the *DES* gene. Overall, we identified 119 variants with the abovementioned characteristics within the *DES* gene, with significant aggregation in the N‐terminal region, particularly in the coil 2B domain of the desmin protein (see Table S1).

**FIGURE 6:**
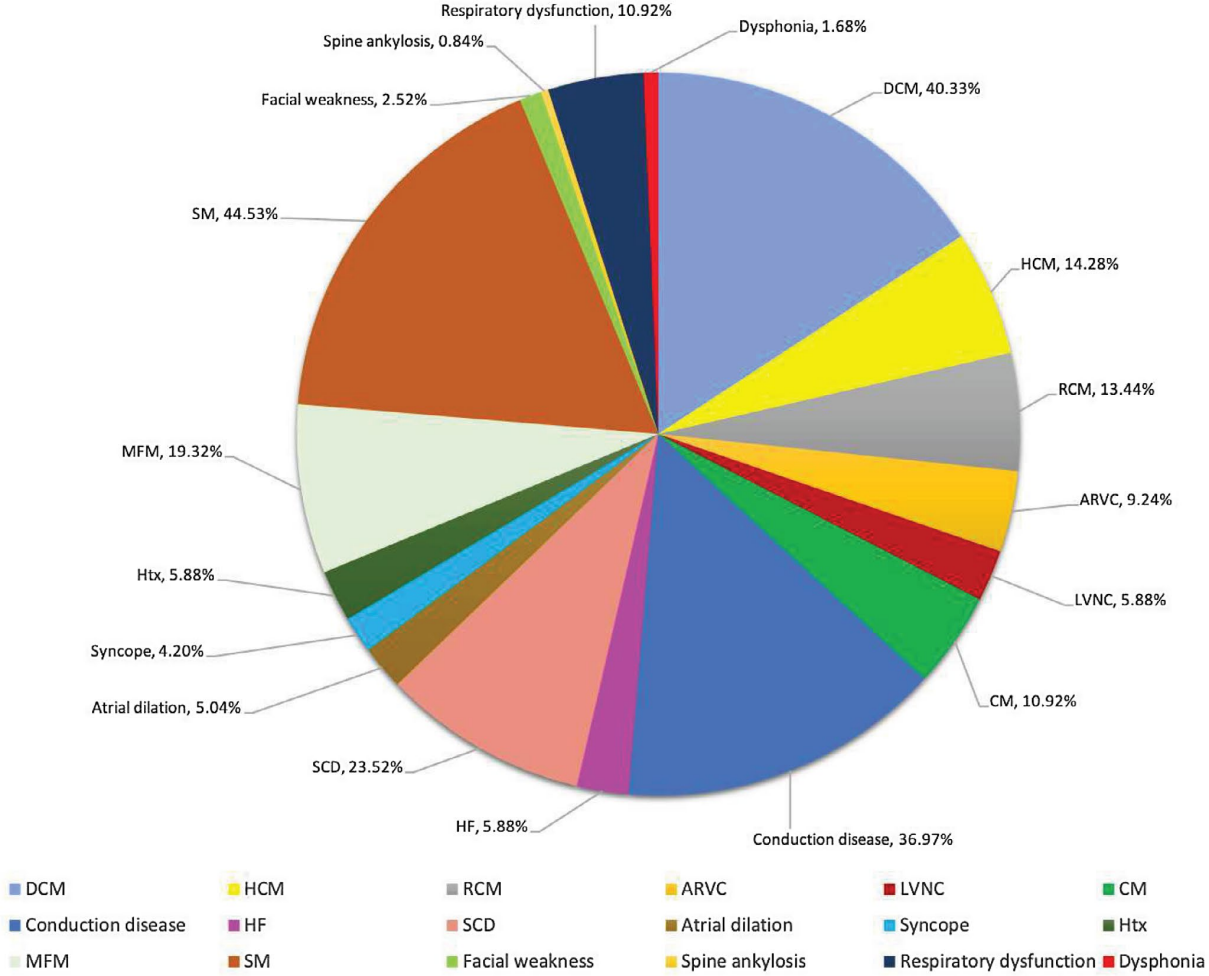
The occurrence frequency of each of the phenotypes is due to missense variants in the *DES* gene.

A total of 18 distinct phenotypes were observed (Figure 6), encompassing a range of cardiomyopathies, such as DCM, HCM, RCM, ARVC, and LVNC. Moreover, various conduction disorders, including arrhythmia, atrial flutter, atrial fibrillation, atrial arrhythmias, short QT syndrome, left bundle branch block, right bundle branch block, bifascicular block, trifascicular block, atrioventricular block, left anterior fascicular block, and atrioventricular block, were identified. Other phenotypes included heart failure, sudden cardiac death, atrial dilation, syncope, heart transplant, myofibrillar myopathy, skeletal myopathy, facial weakness, spine ankylosis, respiratory dysfunction, and dysphonia.

Skeletal myopathy, the most commonly observed phenotype, was reported in 44.5% of individuals harboring missense variants in the *DES* gene across all the desmin domains except linker regions 1, 2, and 3. Among the different types of cardiomyopathies, DCM occurred more frequently, with a 40.3% occurrence rate in patients with missense variants in the desmin gene. Respiratory dysfunctions occurred in 10.9% of the individuals (Figure 6).

**FIGURE 7:**
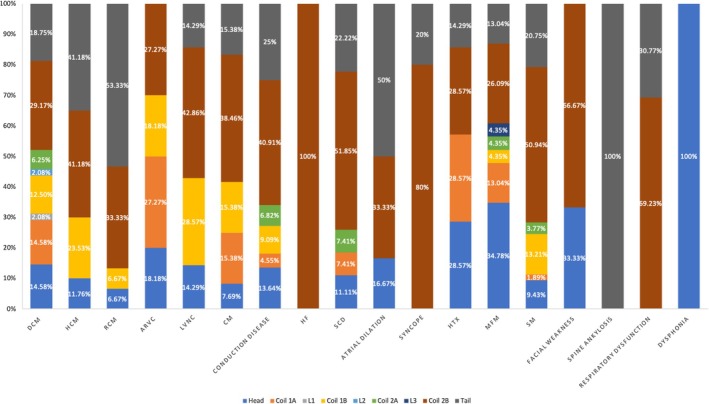
Distribution of occurrence rates of each phenotypic category due to missense variants in different domains of the desmin protein.

DCM was also associated with variants in all the desmin domains except linker 3, and it is worth noting that variants in linkers 1 and 2 are exclusively reported in DCM. Another notable finding is the relatively high incidence rate of various conduction diseases, accounting for 36.9%, resulting from variants in all the desmin domains except the linker regions. Heart failure was predominantly reported among variants located in the coil2B domain in the compiled dataset, while syncope was observed only in patients with variants affecting the second coil2B and tail regions. However, this observation should be interpreted with caution (Figure 7).

Importantly, missense variants in coil1B were not reported in association with end‐stage cardiac phenotypes, such as heart failure, sudden cardiac death, atrial dilation, syncope, or heart transplantation; however, the absence of evidence does not imply absence of association. Spine ankylosis, a rare phenotype linked to desmin, was observed in only one patient with a missense variant in the tail domain. This condition was accompanied by facial weakness, respiratory dysfunction, and generalized myopathy. Respiratory dysfunction was solely caused by missense variants in the N‐terminal region of the desmin protein, particularly affecting coil2B and the tail. Dysphonia, however, has been reported in only two variants found in the desmin head domain (Figure 6) (See Table S1).

These genotype–phenotype observations should be interpreted cautiously. The dataset is literature‐derived and therefore subject to publication bias, selective reporting of severe phenotypes, uneven clinical characterization, and potential co‐variants. Consequently, these findings should be considered hypothesis‐generating rather than definitive correlations.

## 5 Discussion

The *DES* gene is predominantly expressed in skeletal, cardiac, and smooth muscles and plays vital roles in myocyte development, degeneration, and cellular function [48]. Desmin contributes to the stabilization and positioning of mitochondria, and desmin‐related myopathy is associated with mitochondrial dysfunction [47]. As a result, the combination of tissue‐specific changes in the *DES* gene leads to a wide range of clinical phenotypes, including isolated myopathies to different forms of isolated cardiomyopathies and/or cardiac conduction disease. Interestingly, individuals with *DES* gene variants exhibit unique characteristics, such as respiratory insufficiency (severe or chronic), dysphonia, and spine ankylosis [49, 50, 51]. The variability in clinical presentation extends beyond the surface, as family members with the same *DES* gene variant can experience contrasting onset and progression rates. Among carriers, approximately 70% exhibit myopathy. The first neurological signs typically appear around the age of 35 years [7].

Here, we present an Iranian family with HCM harboring a heterozygous variant in the *DES* gene (p.Arg383His). The occurrence of a heterozygous *DES* variant in HCM patients has not been well documented in the literature, making this patient particularly unique [9]. Furthermore, we present a comprehensive table summarizing the clinical characteristics and potential correlations between genotypes and phenotypes within different domains of the desmin protein. This information is based on an extensive search of published and unpublished databases documenting reported carriers of *DES* gene missense variants.

The specific variant p.Arg383His has not been previously reported in individuals affected by *DES*‐related conditions. Although the ClinVar database includes an entry for this variant (Variation ID: 498347), all five patients reported classifying it as a VUS, lacking sufficient evidence to determine its role in disease. However, given that the variant cosegregates in two affected family members, it fulfills ACMG criteria (PM1, PM2, PP2, PP3, and PP1‐supporting) and can therefore be classified as likely pathogenic, representing the probable genetic explanation for the observed cardiac phenotype in this family. Functional validation studies and broader segregation analysis would be required for definitive pathogenic classification. Within the Iranian population, there has been only one other reported patient with a *DES* gene variant resulting in an RCM cardiac phenotype, but that patient was from Germany [3]. In this report, we present an Iranian family that includes two individuals with positive genotype–phenotype correlations, both harboring the same familial variant in the *DES* gene. This family serves as an illustration of the documented clinical heterogeneity observed in patients with desminopathies. The proband (IV‐1) exhibited significant hypertension, an asymmetric left ventricle, and septal hypertrophy, leading to a diagnosis of HOCM at the age of 14 years. Notably, an extremely high value of Pro‐BNP (> 3000 pg/mL) suggested a poor cardiac prognosis for the proband. The presence of biventricular HCM, characterized by RVOT caused by LVH, is a rare occurrence. This condition is correlated with a greater occurrence of both supraventricular and ventricular arrhythmias, severe breathlessness, pulmonary thromboembolism, and deteriorating heart failure and an elevated likelihood of sudden cardiac death [52, 53].

To our knowledge, only one other variant in the *DES* gene (p.Arg454Trp) has been linked to HOCM [54].

The proband's HCM was diagnosed at age 14, consistent with previous studies showing an earlier onset of HCM than of other cardiomyopathies. Skeletal myopathies in individuals carrying variants in the *DES* gene usually appear around the age of 30 years. Furthermore, no neuromuscular disorders were noted in the proband or his family history, and his CK levels were within the normal range. This evidence heavily indicates the occurrence of isolated cardiac phenotypes as a result of this variant. Long‐term monitoring and careful assessment of extracardiac symptoms are essential in managing these patients [52, 53].

Although the inheritance pattern of the desmin gene is autosomal dominant, deviations from this pattern have been documented in a limited number of patients carrying variants of the *DES* gene. In this regard, heterozygous family members carrying a *DES* truncating variant alongside one wild‐type allele did not exhibit any phenotype, indicating a haploinsufficiency pattern [55, 56, 57]. Homozygous missense variants in the *DES* gene have been observed in 6% of patients and are associated with a worse prognosis. These variants are linked to an earlier onset of cardiac disorders, more severe manifestations, and the necessity for end‐stage treatments such as heart transplantation [56, 58]. In rare patients, compound heterozygous or homozygous *DES* truncating variants have been observed, indicating a recessive mode of inheritance [13, 58, 59, 60, 61]. Another exquisite exception was the heterozygous missense compound variant in the *DES* gene, specifically the c.1078G > C (p.A360P) variant, which has shown pathogenic potential when combined with other variants in desmin or other genes, suggesting conditional pathogenicity [62].

The genetic complexity has increased further, as novel trigenic missense variants in *CACNA1C*/*DES*/*MYPN* have been reported in families with HCM, early repolarization, and short QT syndrome [63], as has the co‐occurrence of three distinct missense variants in *DES*/*MYBPC3*/*MYH7* in a single patient [64]. Additionally, *DES* substitution may represent a rare variant that potentially modifies the phenotypic expression of a concomitant *PKP2* variant [5].

In one study, the co‐occurrence of missense variants in the desmin and *LDB3* genes was associated with the development of LVNC [65]. Furthermore, two missense variants in the tail region of desmin, one with myotilin and the other with laminA, have been reported as associated variants in affected patients [57, 66].

One patient with the p.Arg644Cys variant in lamin A/C and the p.Val469Met variant in the desmin tail region experienced severe muscle weakness and complete heart block, necessitating heart transplantation. Notably, 40% of *DES* variants occur de novo, particularly in the segment of the gene encoding the 2B helical region, indicating a hotspot for variants [67].

Genotype–phenotype correlation involves not only the inheritance pattern and molecular pathogenic mechanism of the variant but also the specific location of the variant within the desmin domains, contributing to phenotypic variability. A meta‐analysis performed by van Spaendonck‐Zwarts et al. examined 40 different *DES* gene variants and revealed that variants in the 2B domain were primarily associated with isolated neurological phenotypes, whereas variants in the head or tail domains were commonly associated with cardiac phenotypes [13].

In contrast, the data collected in this study do not support a strong genotype–phenotype correlation. Cardiac and myopathy phenotypes are more widespread across domains than previously believed (see Table S1). Although certain conditions, such as heart failure, are exclusively associated with variants in the second coil2B, missense variants in the coil1B domain do not exhibit any connection with end‐stage cardiac phenotypes, such as heart failure, sudden cardiac death, atrial dilation, syncope, or heart transplantation. Similarly, respiratory dysfunction is caused solely by missense variants in the N‐terminal region of the desmin protein, affecting coil2B and the tail, while dysphonia has been reported in only two variants within the desmin head domain. However, these observations should be interpreted with caution due to limited confidence (see Table S1).

Next‐generation sequencing (NGS) technology and bioinformatics have revolutionized the development of cost‐effective and accurate diagnostic tools for genetic disorders, including cardiomyopathies [68]. WGS analysis of intronic regions and the mitochondrial genome can also be valuable for identifying pathogenic variants, particularly in 9% of HCM patients with a familial history but no causative variant detected during initial genetic testing [16]. Identifying such variants prompted genetic evaluation and cascade screening of the patient's family, leading to the discovery of the patient's mother as a carrier with compatible clinical manifestations. Intergender phenotypic variability in desmin‐related diseases highlights the significance of precise genetic analysis to avoid false negative results. Consequently, a comprehensive characterization of the phenotype, incorporating WGS and complementary analysis of copy number variations, can provide optimal care for affected families and enable appropriate planning for future generations.

It is important to acknowledge certain limitations of our study. These limitations include the inability to perform histologic and electron microscopic analyses of affected muscle tissues in our case series, the limitation of segregation analysis to two affected individuals, the limitation of formal assessment of disease penetrance, as well as limited access to complete clinical records of all family members, and the challenges of following up all individuals due to the occurrence of late‐onset phenotypes. Notably, our investigation focused exclusively on missense variants and compiled emerging phenotypes mentioned in the literature at a specific time point. These limitations constrain definitive pathogenic classification and limit interpretation of intrafamilial phenotypic variability. Consequently, the genotype–phenotype suggestions in our study can be reasonably relied upon to a certain extent.

## Author Contributions

**Saeideh Kavousi:** data curation, formal analysis, investigation, methodology, writing – original draft. **Farzad Kamali:** resources, validation, visualization. **Bahareh Rabbani:** data curation, resources, software, validation. **Mehrdad Behmanesh:** software, validation. **Nejat Mahdieh:** conceptualization, project administration, resources, supervision, writing – review and editing. **Mehrdad Noruzinia:** conceptualization, funding acquisition, project administration, supervision, validation.

## Funding

The authors have nothing to report.

## Ethics Statement

The study involving human participants was carried out according to the ethical standards set by the Ethics Committee of the Tarbiat Modares University, Tehran, Iran (Approval ID: IR.MODARES.REC.1399.253). The study adhered to the ethical principles of the World Medical Association Declaration of Helsinki. Informed consent was obtained from the family for the genetic analysis, which was conducted in compliance with national ethics regulations.

## Consent

A written informed consent was obtained from the patient/patients.

## Conflicts of Interest

The authors declare no conflicts of interest.

## Supporting information


**Table S1:** Overview of the missense variants reported in the *DES* gene.

## Data Availability

The datasets generated and/or analyzed during the current study are available in the ClinVar repository [https://www.ncbi.nlm.nih.gov/clinvar/variation/498347/]. The accession number of the variant in ClinVar is as follows: NM_001927.4 (DES): c.1148G>A (p.Arg383His): SCV004100693.1. Additionally, interested parties may request access to the data from the corresponding author, and reasonable requests will be accommodated. However, due to confidentiality concerns related to patient information, the data cannot be shared.
