# Supplementary material for: Unveiling New Insights: Reinterpreting DES Mutation, p.Arg383His, Through a Study of an Iranian Family With Isolated Hypertrophic Cardiomyopathy, Implication for Phenotype–Genotype Correlation Analysis
Source: Clin Case Rep. 2026 Jul 28;14(8):e73051. doi: 10.1002/ccr3.73051 (PMC13415748; doi:10.1002/ccr3.73051)
Supplement: Supplementary file 1 — Table S1: Overview of the missense variants reported in the DES gene. [file CCR3-14-e73051-s001.docx]

**Supplemental Table 1.** Overview of the missense variants reported in the *DES* gene.

| No | Variants | | rs# | Varsome  (ACMG Categorization) | Phenotype | | | | | | | | | | | | | | | | | | Clinvar  Submissions | References |
| --- | --- | --- | --- | --- | --- | --- | --- | --- | --- | --- | --- | --- | --- | --- | --- | --- | --- | --- | --- | --- | --- | --- | --- | --- |
|  | **DNA level** | **Protein level** |  | | **DCM** | **HCM** | **RCM** | **ARVC** | **LVNC** | **CM** | **Conduction disease** | **HF** | **SCD** | **Atrial dilation** | **Syncope** | **Htx** | **MFM** | **SM** | **Facial weekness** | **Spine ankylosis** | **Respiratory dysfunction** | **Dysphonia** |  | |
| 1 | c.1A>G | p.Met1Val | rs1057523274 | P |  |  |  |  |  |  |  |  |  |  |  |  | + |  |  |  |  |  | LP:3  VCV000388926.15 | ^1^ |
| 2 | c.2T>C | p.Met1Thr | - | P |  |  |  |  |  |  |  |  |  |  |  |  | + |  |  |  |  |  | LP:1  VCV001066470.6 | ^1^ |
| 3 | c.5G>T | p.Ser2Ile | rs58999456 | VUS/LP |  | + |  |  |  |  |  |  |  |  |  | + | + |  |  |  |  |  | VUS:1  VCV000066414.2 | ^2–4^ |
| 4 | c.17C>G | p.Ser6Trp | - | VUS | + |  |  |  |  |  | + |  |  |  |  |  | + | + |  |  |  | + | VUS:2  VCV001436615.4 | ^5,6^ |
| 5 | c.20C>T | p.Ser7Phe | rs903985237 | VUS/LP |  |  |  |  |  |  |  |  |  |  |  |  |  |  |  |  |  | + | VUS:1  VCV000594969.4 | ^7^ |
| 6 | c.35C>T | p.Ser12Phe | rs267607495 | P | + |  |  |  |  |  | + |  | + |  |  |  |  | + | + |  |  |  | P:2  LP:2  VCV000066412.15 | ^1,8^ |
| 7 | c.38C>T | p.Ser13Phe | rs62636495 | P | + |  |  |  |  |  |  |  |  |  |  |  |  |  |  |  |  |  | P:9  VCV000044260.22 | ^2,9–15^ |
| 8 | c.38C>A | p.Ser13Tyr | rs62636495 | LP | + | + |  | + |  |  | + |  |  |  |  |  | + | + |  |  |  |  | VUS:3  VCV000541304.10 | ^10–14,16^ |
| 9 | c.46C>T* | p.Arg16Cys | rs60798368 | LP |  |  | + |  |  |  | + |  |  | + |  | + |  |  |  |  |  |  | P:1  VCV000066413.3 | ^2,17,18^ |
| 10 | c.72C>G | p.Phe24Leu | - | VUS |  |  |  | + |  |  |  |  |  |  |  |  |  |  |  |  |  |  | - | ^19^ |
| 11 | c.106C>T | p.Pro36Ser | - | LB |  |  |  |  |  |  | + |  |  |  |  |  |  |  |  |  |  |  | VUS:1  VCV002073688.1 | ^20^ |
| 12 | c.127A>G | p.Lys43Glu | rs397516689 | VUS/ LP | + |  |  |  |  |  |  |  |  |  |  |  |  |  |  |  |  |  | VUS:1  VCV000044250.4 | ^19,21^ |
| 13 | c.130G>A | p.Gly44Ser | rs1064794869 | LP |  |  |  |  |  |  |  |  |  |  |  |  |  |  |  |  |  |  | LP:1  VCV000421043.2 | ^22^ |
| 14 | c.137C>T | p.Ser46Phe | rs60794845 | LP |  |  |  |  |  |  |  |  |  |  |  |  | + |  |  |  |  |  | - | ^2,3,23^ |
| 15 | c.137C>A | p.Ser46Tyr | rs60794845 | P |  |  |  |  |  |  |  |  |  |  |  |  | + |  |  |  |  |  | LP:1  VCV000066405.5 | ^2,3^ |
| 16 | c.155G>T | p.Arg52Leu | - | VUS/ LP |  |  |  |  |  |  |  |  |  |  |  |  |  | + |  |  |  |  | - | ^24^ |
| 17 | c.170C>T | p.Ser57Leu | rs372825868 | VUS/ LP | + |  |  |  |  | + |  |  | + |  |  |  | + | + |  |  |  |  | VUS:12  LB:1  B:1  VCV000044254.36 | ^21,25–28^ |
| 18 | c.193G>A | p.Gly65Ser | rs397516692 | LB | + |  |  |  |  |  |  |  |  |  |  |  |  |  |  |  |  |  | LP:1  VUS:7  LB:1  B:1  VCV000044256.22 | ^19,29^ |
| 19 | c.206T>C | p.Leu69Pro | rs1200347906 | VUS |  |  |  |  | + |  |  |  |  |  |  |  |  |  |  |  |  |  | VUS:3  VCV001785367.4 | ^30^ |
| 20 | c.230C>T | p.Thr77Ile | rs1392710887 | VUS/LP |  |  |  |  |  |  | + |  | + |  |  |  |  |  |  |  |  |  | VUS:1  VCV001447427.3 | ^20^ |
| 21 | c.322G>A | p.Glu108Lys | rs62636490 | LP | + |  |  |  |  |  | + |  |  |  |  |  |  |  |  |  |  |  | VUS:3  VCV000066408.9 | ^31^ |
| 22 | c.343C>A | p.Leu115Ile | - | LP | + |  |  |  |  | + |  |  |  |  |  |  |  |  |  |  |  |  | P:1  VCV002169507.1 | ^32,33^ |
| 23 | c.347A>T | p.Asn116Ile | rs267607499 | P |  |  |  | + |  |  |  |  |  |  |  |  |  |  |  |  |  |  | LP:1  VUS:7  VCV000228547.9 | ^9,34,35^ |
| 24 | c.347A>G | p.Asn116Ser | rs267607499 | P |  |  |  | + |  |  |  |  |  |  |  | + |  | + |  |  |  |  | P:1  Likely Pathogenic:2  VCV000066411.13 | ^9,34–37^ |
| 25 | c.358G>C | p.Ala120Pro | rs794728996 | P |  |  |  |  |  |  |  |  |  |  |  |  | + |  |  |  |  |  | P:1  VUS:1  VCV000201726.7 | ^38^ |
| 26 | c.359C>A | p.Ala120Asp | - | P |  |  |  |  |  | + | + |  | + |  |  | + |  |  |  |  |  |  | VUS:1  VCV000854289.8 | ^39^ |
| 27 | c.364T>G | p.Tyr122Asp | rs794728994 | P |  |  |  |  |  |  |  |  |  |  |  |  |  |  |  |  |  |  | LP:1  VCV000201721.2 | ^40^ |
| 28 | c.364T>C | p.Tyr122His |  | LP |  |  | + |  |  |  |  |  | + |  |  |  |  |  |  |  |  |  |  |  |
| 29 | c.365A>G | p.Tyr122Cys | rs1400593451 | P |  |  |  | + |  |  |  |  |  |  |  |  | + |  |  |  |  |  | LP:2  VUS:2  VCV000618590.14 | ^19,40^ |
| 30 | c.376G>A | p.Val126Met | - | LP | + |  |  |  |  |  |  |  |  |  |  |  |  |  |  |  |  |  | LP:1  VCV000977180.1 | ^41^ |
| 31 | c.376G>T | p.Val126Leu | rs876657770 | LP | + |  |  |  |  |  |  |  |  |  |  |  |  |  |  |  |  |  | VUS:2  VCV000228549.8 | ^42^ |
| 31 | c.380G>C | p.Arg127Pro | rs397516694 | P | + |  |  |  |  |  |  |  | + |  |  |  |  |  |  |  |  |  | LP:2  VUS:2  VCV000044259.14 | ^43^ |
| 33 | c.407T>C | p.Leu136Pro | rs397516695 | P | + |  |  |  |  |  |  |  |  |  |  |  |  |  |  |  |  |  | LP:3  VCV000265811.5 | ^44^ |
| 34 | c.407T>A | p.Leu136His | rs397516695 | LP | + |  |  |  |  |  |  |  |  |  |  |  | + |  |  |  |  |  | VUS:10  VCV000044261.25 | ^29,45–50^ |
| 35 | c.449G>C | p.Arg150Pro | - | VUS/ LP | + |  |  |  |  |  |  |  |  |  |  |  |  |  |  |  |  |  | - | ^19^ |
| 36 | c.603G>T | p.Lys201Asn | - | VUS/ LP | + |  |  |  |  |  |  |  |  |  |  |  |  |  |  |  |  |  | - | ^51^ |
| 37 | c.629C>A | p.Ala210Asp | - | LP |  |  |  |  | + |  |  |  |  |  |  |  |  | + |  |  |  |  | - | ^21^ |
| 38 | c.635G>A | p.Arg212Gln | rs144261171 | VUS | + |  |  |  | + | + |  |  |  |  |  |  |  |  |  |  |  |  | VUS:13  LB:1  B:1  VCV000178015.34 | ^27,30,37,52,53^ |
| 39 | c.638C>T | p.Ala213Val | rs41272699 | LB | + |  | + | + |  | + |  |  |  |  |  |  | + |  |  |  |  |  | P:1  VUS:1  LB:8  B:14  VCV000044265.45 | ^18,54–56,56,56–69^ |
| 40 | c.641A>T | p.Asp214Val | - | LP |  |  |  |  |  |  |  |  |  |  |  |  |  | + |  |  |  |  | - | ^54^ |
| 41 | c.640G>T* | p.Asp214Tyr | - | P |  |  |  |  |  |  | + |  |  |  |  |  |  | + |  |  |  |  | VUS:1  VCV001029548.2 | ^70^ |
| 42 | c.655A>C* | p.Thr219Pro | - | VUS/ LP |  | + |  |  |  |  |  |  |  |  |  |  |  | + |  |  |  |  | - | ^71^ |
| 43 | c.665G>A | p.Arg222His | rs367961979 | VUS | + |  |  |  |  |  |  |  |  |  |  |  |  |  |  |  |  |  | VUS:5  LB:6  B:1  VCV000178016.22 | ^51,72,73^ |
| 44 | c.679C>T | p.Arg227Cys | rs767743962 | LP | + |  |  |  |  |  |  |  |  |  |  |  |  |  |  |  |  |  | VUS:2  VCV000411141.7 | ^74^ |
| 45 | c.700G>A* | p.Glu234Lys | rs774739275 | VUS/ LP |  | + |  |  |  |  | + |  |  |  |  |  |  |  |  |  |  |  | VUS:1  VCV000411150.4 | ^75^ |
| 46 | c.721A>G* | p.Lys241Glu | rs201945924 | VUS/ LP |  |  |  | + |  |  |  |  |  |  |  |  |  |  |  |  |  |  | - | ^56^ |
| 47 | c.735G>C | p.Glu245Asp | rs267607486 | P |  | + |  |  |  |  | + |  |  |  |  |  |  | + |  |  |  |  | P:1  LP:2  VCV000066420.8 | ^76–78^ |
| 48 | c.735G>T | p.Glu245Asp |  | P |  | + |  |  |  |  | + |  |  |  |  |  |  | + |  |  |  |  | - | ^4,23,36,79,80^ |
| 49 | c.738G>T | p.Glu246Asp | - | LP | + |  |  |  |  |  |  |  |  |  |  |  |  | + |  |  |  |  | - | ^54^ |
| 50 | c.785A>T | p.Glu262Val | rs147327878 | VUS | + |  |  |  |  |  |  |  |  |  |  |  |  |  |  |  |  |  | VUS:5  LB:3  B:1  VCV000044270.23 | ^29,37^ |
| 51 | c.821T>G | p.Leu274Arg | rs267607494 | LP |  |  |  |  |  |  | + |  |  |  |  |  |  | + |  |  |  |  | VUS:1  VCV000066422.4 | ^1^ |
| 52 | c.821T>C | p.Leu274Pro | rs267607494 | LP |  |  |  |  |  |  | + |  | + |  |  |  |  | + |  |  |  |  | - | ^1,8,24^ |
| 53 | c.823A>G | p.Arg275Gly | rs994389035 | VUS/ LP | + |  |  |  |  |  |  |  |  |  |  |  |  |  |  |  |  |  | VUS:2  VCV001368704.6 | ^32,42^ |
| 54 | c.832C>T | p.Arg278Trp | rs794728985 | LP | + |  |  |  |  |  |  |  |  |  |  |  |  |  |  |  |  |  | VUS:3  VCV000201703.10 | ^19,81^ |
| 55 | c.854C>T | p.Ala285Val | rs1368507241 | LP | + |  |  |  |  |  | + |  | + |  |  |  | + |  |  |  |  |  | LP:1  VUS:1  VCV000915758.6 | ^82,83^ |
| 56 | c.883T>G | p.Trp295Gly | rs794728986 | LP |  |  |  |  |  |  |  |  |  |  |  |  | + |  |  |  |  |  | LP:1  VUS:1  VCV000201704.8 | ^84^ |
| 57 | c.893C>T | p.Ser298Leu | rs62636491 | LP | + |  |  |  |  |  | + |  | + |  |  |  | + |  |  |  |  |  | LP:1  VUS:7  B:2 | ^37,63,83,85,86^ |
| 58 | c.934G>A | p.Asp312Asn | rs34337334 | VUS | + |  |  |  |  |  |  |  | + |  |  |  |  | + |  |  |  |  | VUS:13  LB:1  B:1  VCV000044274.42 | ^29,31,50,63,67,87,88^ |
| 59 | c.935A>C* | p.Asp312Ala | rs148947510 | VUS |  | + |  |  |  |  |  |  | + |  |  |  |  |  |  |  |  |  | VUS:4  LB:3  VCV000044275.24 | ^27,53,89^ |
| 60 | c.976C>T | p.His326Tyr | rs794728987 | LP |  |  |  | + |  |  |  |  |  |  |  |  |  |  |  |  |  |  | LP:1  VUS:2  VCV000201705.11 | ^39^ |
| 61 | c.991T>A | p.Tyr331Asn | rs1064795298 | VUS/ LP |  |  |  | + |  |  |  |  |  |  |  |  |  |  |  |  |  |  | VUS:2  VCV000421689.5 | ^19^ |
| 62 | c.1006G>T | p.Asp336Tyr |  | VUS/ LP | + |  |  |  |  |  | + |  |  |  |  |  |  | + |  |  |  |  | - | ^4^ |
| 63 | c.1009G>A | p.Ala337Thr | rs59962885 | VUS/ LP |  |  |  |  |  | + |  |  | + |  |  |  |  | + |  |  |  |  | VUS:4  VCV000382176.9 | ^20,90^ |
| 64 | c.1009G>C | p.Ala337Pro | rs59962885 | P | + |  |  |  |  |  | + |  |  |  |  |  |  | + |  |  |  |  | P:2  VCV000016820.3 | ^61,90^ |
| 65 | c.1013T>G | p.Leu338Arg | rs57496341 | P |  |  |  |  |  |  |  |  |  |  |  |  | + | + |  |  | + |  | P:1  Likely Pathogenic:1  VCV000066387.9 | ^61,91^ |
| 66 | c.1013T>C | p.Leu338Pro | rs57496341 | P |  |  |  |  |  | + |  |  |  |  |  |  | + |  |  |  |  |  | LP:1  VCV000239071.6 | ^61^ |
| 67 | c.1019G>A | p.Gly340Asp | rs1559353118 | VUS/ LP | + |  |  |  |  |  |  |  |  |  |  |  |  |  |  |  |  |  | VUS:2  VCV000574314.7 | ^19^ |
| 68 | c.1024A>G | p.Asn342Asp | rs267607482 | P |  |  |  |  |  |  | + | + |  |  |  |  |  | + |  |  |  |  | P:5  VCV000066388.13 | ^4,9,66,92–94^ |
| 69 | c.1030T>C | p.Ser344Pro | rs886044226 | LP |  |  |  |  |  |  |  |  |  |  |  |  |  | + |  |  |  |  | VUS:3  VCV000289633.6 | ^24^ |
| 70 | c.1034T>C | p.Leu345Pro | rs57639980 | P | + |  |  |  |  |  | + |  |  |  |  |  | + | + |  |  |  |  | P:4  LP:1  VCV000016825.14 | ^4,95–98^ |
| 71 | c.1043A>C | p.Gln348Pro | rs1411703397 | P |  |  |  |  |  |  |  |  |  |  |  |  |  | + |  |  |  |  | P:1  VCV000617786.2 | ^99^ |
| 72 | c.1048C>T | p.Arg350Trp | rs62636492 | P | + |  |  |  |  |  |  |  |  |  |  |  |  |  |  |  |  |  | P:2  LP:1  VUS:3  VCV000044244.12 | ^20,63,87,100^ |
| 73 | c.1049G>C | p.Arg350Pro | rs57965306 | P |  | + |  |  |  |  | + | + |  |  |  |  |  | + |  |  | + |  | P:5  VCV000016835.25 | ^67,101,101–103,103–106^ |
| 74 | c.1049G>A | p.Arg350Gln | rs57965306 | LP |  | + |  |  |  |  |  |  | + |  |  |  |  | + |  |  |  |  | VUS:5  VCV000596421.13 | ^107,108^ |
| 75 | c.1055T>C | p.Leu352Ser | rs775085773 | LP |  |  |  |  |  |  |  |  |  |  |  |  |  |  |  |  |  |  | LP:1  VCV000947073.5 | ^109^ |
| 76 | c.1064G>C | p.Arg355Pro | rs61368398 | LP |  |  |  |  |  |  | + |  | + | + | + |  |  | + |  |  |  |  | LP:1  VUS:3  VCV000066389.12 | ^4,110^ |
| 77 | c.1069G>C | p.Ala357Pro | rs58898021 | P |  |  |  |  |  |  |  |  |  |  |  |  | + | + |  |  |  |  | P:2  VCV000066390.6 | ^18,111–113^ |
| 78 | c.1078G>T* | p.Ala360Ser | - | LP |  |  |  |  | + |  |  |  |  |  |  |  |  | + |  |  |  |  | VUS:3  VCV001517261.6 | ^30^ |
| 79 | c.1078G>C* | p.Ala360Pro | rs121913000 | P |  |  | + |  |  |  | + |  |  |  |  |  |  | + |  |  | + |  | P:1  VCV000016821.2 | ^61,71,90,114,115^ |
| 80 | c.1099A>T | p.Ile367Phe | rs62636494 | LP |  | + | + |  |  |  | + |  | + |  | + |  |  | + |  |  | + |  | - | ^116^ |
| 81 | c.1109T>C | p.Leu370Pro | rs59308628 | P | + |  |  |  |  |  | + |  | + |  |  |  | + | + |  |  | + |  | P:3  VCV000066393.11 | ^24,67,112,113,117–120^ |
| 82 | c.1130T>C | p.Leu377Pro | rs1432061016 | LP |  |  |  |  |  |  |  |  |  |  |  |  |  | + |  |  | + |  | LP:1  VCV001471108.5 | ^80^ |
| 83 | **c.1148G>A**** | **p.Arg383His** | rs1292042317 | LP |  | + |  |  |  |  |  | + |  |  |  |  |  |  |  |  |  |  | VUS:5  VCV000498347.13 | This study |
| 84 | c.1151A>G | p.His384Arg | rs1553603566 | LP |  |  |  |  |  |  |  |  |  |  |  |  |  |  |  |  |  |  | LP:1  VCV000522692.4 | ^121^ |
| 85 | c.1154T>C | p.Leu385Pro | rs57955682 | P | + |  |  |  |  |  | + | + |  |  |  |  |  | + |  |  |  |  | P:1  VCV000016829.4 | ^122^ |
| 86 | c.1157G>A | p.Arg386His | rs1029457073 | LP | + |  |  |  |  |  |  |  |  |  |  |  |  |  |  |  |  |  | VUS:3  VCV001284539.5 | ^123^ |
| 87 | c.1166A>C | p.Gln389Pro | rs121913004 | P |  |  |  |  |  | + | + |  |  |  |  |  |  | + |  |  |  |  | P:1 | ^112,124^ |
| 88 | c.1175T>C | p.Leu392Pro | rs62636493 | LP |  | + | + |  |  |  |  |  | + |  |  |  |  | + |  |  |  |  | VUS:1  VCV000066395.4 | ^36,116,119^ |
| 89 | c.1178A>T | p.Asn393Ile | rs121913001 | LP |  |  | + |  |  | + | + | + | + |  | + |  |  | + |  |  | + |  | P:1  VUS:1  VCV000016822.5 | ^58,61,64,90,93^ |
| 90 | c.1193T>C | p.Leu398Pro | rs796115330 | LP |  |  |  |  | + |  |  |  |  |  |  |  |  |  |  |  |  |  | VUS:1  VCV000960484.8 | ^125^ |
| 91 | c.1195G>T | p.Asp399Tyr | rs61130669 | P | + |  |  |  |  |  | + |  | + |  |  |  |  | + |  |  |  |  | LP:1  VCV000066396.4 | ^36,61,112^ |
| 92 | c.1201G>A | p.Glu401Lys | rs57694264 | P |  |  |  |  |  | + | + |  |  |  |  |  |  | + |  |  |  |  | LP:1  VCV000066397.9 | ^61,112^ |
| 93 | c.1202A>G | p.Glu401Gly | - | P | + |  |  |  |  |  |  |  |  |  |  |  |  |  |  |  |  |  | LP:1  VCV000969855.7 | ^27^ |
| 94 | c.1203G>C | p.Glu401Asp | - | P | + |  |  | + |  | + | + | + | + |  |  | + |  | + |  |  |  |  | P:1  VUS:1  VCV001324221.4 | ^126,127^ |
| 95 | c.1205T>A | p.Ile402Asn | - | LP | + |  |  |  |  |  | + | + | + |  |  |  |  | + | + |  | + |  | - | ^5^ |
| 96 | c.1214A>C | p.Tyr405Ser | - | LP |  |  |  |  |  |  |  |  |  |  |  |  |  |  |  |  |  |  | LP:1  VCV001501730.3 | ^128^ |
| 97 | c.1216C>T | p.Arg406Trp | rs121913003 | P |  | + | + |  |  |  | + | + | + | + | + | + |  | + | + |  | + |  | P:8  LP:1  VCV000016826.24 | ^4,17,92,93,112,129–133^ |
| 98 | c.1217G>C | p.Arg406Pro | - | P |  |  |  |  |  |  |  |  |  |  |  |  |  |  |  |  |  |  | LP:1  VCV002109936.1 | ^134^ |
| 99 | c.1222C>G | p.Leu408Val | - | LP |  |  |  |  | + |  |  |  |  |  |  |  |  |  |  |  |  |  | - | ^125^ |
| 100 | c.1237G>A | p.Glu413Lys | rs61726467 | P |  |  | + |  |  |  | + |  | + |  |  |  |  |  |  |  |  |  | P:1  LP:1  VCV000066398.8 | ^67,104,112,132,135–137^ |
| 101 | c.1237_1238delGAinsCG | p.Glu413Arg | - | LP |  | + |  |  |  |  | + |  |  |  |  |  |  | + |  |  |  |  | - | ^4^ |
| 102 | c.1243_1244delCGinsGA | p.Arg415Glu | - | VUS/ LP | + |  | + |  |  |  |  |  | + |  |  |  |  | + |  |  |  |  | - | ^116^ |
| 103 | c.1243C>T | p.Arg415Trp | rs751942358 | LP |  |  |  |  |  |  |  |  |  |  |  |  |  | + |  |  |  |  | VUS:6  VCV000201709.21 | ^67,138^ |
| 104 | c.1244G>A | p.Arg415Gln | rs1262288015 | P |  |  |  |  | + |  |  |  |  |  |  |  |  |  |  |  |  |  | VUS:3  VCV000626714.9 | ^139^ |
| 105 | c.1255C>T | p.Pro419Ser | rs62635763 | P | + | + | + |  |  |  | + |  |  | + |  |  | + | + |  |  |  |  | P:3  LP:1  VCV000039718.13 | ^4,7,36,116,140,140–142^ |
| 106 | c.1297C>A | p.Pro433Thr | rs869025381 | VUS/ LP | + |  | + |  |  |  |  |  |  |  |  |  |  |  |  |  |  |  | VUS:1  VCV000222544.1 | ^143^ |
| 107 | c.1315G>A | p.Glu439Lys | rs1114167347 | P | + |  |  |  |  |  | + |  |  |  |  |  |  | + |  |  | + |  | VUS:2  VCV000265838.2 | ^4^ |
| 108 | c.1325C>T | p.Thr442Ile | rs121913005 | P | + |  |  |  |  | + | + |  | + |  |  |  |  | + |  |  | + |  | P:4  VCV000016834.24 | ^4,66,112,136,144^ |
| 109 | c.1333A>G | p.Thr445Ala | rs267607498 | VUS |  |  |  |  |  |  |  |  |  |  |  |  |  | + |  | + | + |  | VUS:4  VCV000066399.11 | ^1,67,145^ |
| 110 | c.1346A>T | p.Lys449Met | - | P |  |  |  |  |  |  |  |  |  |  |  |  |  |  |  |  |  |  | - | - |
| 111 | c.1346A>C | p.Lys449Thr | rs267607485 | P |  |  |  |  |  |  |  |  |  |  |  |  | + |  |  |  |  |  | P:3  LP:2  VCV000066400.19 | ^3,18,32,112,119,136^ |
| 112 | c.1353C>G | p.Ile451Met | rs121913002 | LP | + |  |  |  |  |  |  |  |  |  |  |  |  |  |  |  |  |  | P:1  LP:2  VUS:6  B:1  VCV000016824.17 | ^27,92,93,95,112,136,146–150^ |
| 113 | c.1358C>T | p.Thr453Ile | rs267607488 | P |  |  | + |  |  |  | + |  |  | + |  |  |  |  |  |  |  |  | LP:1  VUS:3  VCV000066401.11 | ^17,23,112,138,151^ |
| 114 | c.1360C>T* | p.Arg454Trp | rs267607490 | P | + | + | + |  |  | + | + |  | + | + | + | + | + | + |  |  | + |  | P:6  LP:4  VUS:1  VCV000066402.22 | ^4,5,7,9,10,94,104,129,136,144,152–159^ |
| 115 | c.1361G>A | p.Arg454Gln | rs541585670 | LP | + |  |  |  |  |  |  |  |  |  |  |  |  |  |  |  |  |  | VUS:3  VCV000191616.8 | ^42^ |
| 116 | c.1370A>T | p.Glu457Val | rs267607496 | P |  |  | + |  |  |  | + |  | + |  |  |  |  | + |  |  |  |  | - | ^1^ |
| 117 | c.1375G>A | p.Val459Ile | rs73991549 | VUS | + |  |  |  |  |  | + |  |  |  |  |  |  |  |  |  |  |  | LB:13 B:7  VCV000044252.41 | ^63,87,112,136,160,161^ |
| 118 | c.1379G>T | p.Ser460Ile | rs267607491 | LP |  |  | + |  |  |  | + |  | + |  |  |  |  | + |  |  |  |  | - | ^112^ |
| 119 | c.1405G>A* | p.Val469Met | rs267607487 | VUS |  | + |  |  |  |  | + |  |  |  |  |  |  | + |  |  |  |  | - | ^112,136,162^ |

P = Pathogenic; LP = Likely pathogenic; VUS = Genetic variant of unknown significances; DCM = Dilated cardiomyopathy; HCM = Hypertrophic cardiomyopathy; RCM = Restrictive cardiomyopathy; ARVC = Arrhythmogenic right ventricular cardiomyopathy; LVNC = Left ventricular non-compaction cardiomyopathy; CM = Cardiomyopathy; HF = Heart failure; SCD = Sudden cardiac death; HTx = Heart transplantation; MFM = Myofibrillar myopathy; SM = Skeletal myopathy.

**Comments:**

The variants with a * sign have discrepancies with the autosomal dominant inheritance pattern and the heterozygosity of the mutated allele:

p.Arg16Cys. Homozygous

p.Asp214Tyr. Homozygous

p.Thr219Pro. Homozygous

p.Glu234Lys. Trigenic, *MYPN*-p.R989H/*CACNA1C* -p.R1973P

p.Lys241Glu. *PKP2*-p.T816RfsX10

p.Asp312Ala. *MYBPC3*-p.R1002W, *MYH7*-p.D43N

p.Ala360Ser. *LDB3*-p.I615N

p.Ala360Pro. Compound heterozygous, *DES*-p.N393I

p.Arg454Trp. *MYOT*-p.Q74K

p.Val469Met. *LMNA*-p.R644C

The variant with a ** sign is the one studied in this article.

**Reference:**

1. Hong, D. *et al.* A series of Chinese patients with desminopathy associated with six novel and one reported mutations in the desmin gene. *Neuropathol Appl Neurobiol* **37**, 257–270 (2011).

2. Sharma, S., Mücke, N., Katus, H. A., Herrmann, H. & Bär, H. Disease mutations in the ‘head’ domain of the extra-sarcomeric protein desmin distinctly alter its assembly and network-forming properties. *J Mol Med (Berl)* **87**, 1207–1219 (2009).

3. Selcen, D., Ohno, K. & Engel, A. G. Myofibrillar myopathy: clinical, morphological and genetic studies in 63 patients. *Brain* **127**, 439–451 (2004).

4. Wahbi, K. *et al.* High cardiovascular morbidity and mortality in myofibrillar myopathies due to DES gene mutations: a 10-year longitudinal study. *Neuromuscul Disord* **22**, 211–218 (2012).

5. Weihl, C. C. *et al.* Autophagic vacuolar pathology in desminopathies. *Neuromuscul Disord* **25**, 199–206 (2015).

6. Carroll, L. S. *et al.* Desminopathy presenting as late onset bilateral facial weakness, with diagnosis supported by lower limb MRI. *Neuromuscul Disord* **31**, 249–252 (2021).

7. Vattemi, G. *et al.* Clinical, morphological and genetic studies in a cohort of 21 patients with myofibrillar myopathy. *Acta Myol* **30**, 121–126 (2011).

8. Hong, D. *et al.* [Clinical characteristics and desmin mutations in patients with desminopathy associated cardiomyopathy from 5 Chinese families]. *Zhonghua Xin Xue Guan Bing Za Zhi* **38**, 420–424 (2010).

9. Brodehl, A. *et al.* Dual color photoactivation localization microscopy of cardiomyopathy-associated desmin mutants. *J Biol Chem* **287**, 16047–16057 (2012).

10. van Spaendonck-Zwarts, K. Y. *et al.* Genetic analysis in 418 index patients with idiopathic dilated cardiomyopathy: overview of 10 years’ experience. *Eur J Heart Fail* **15**, 628–636 (2013).

11. McCormick, E. M., Kenyon, L. & Falk, M. J. Desmin common mutation is associated with multi-systemic disease manifestations and depletion of mitochondria and mitochondrial DNA. *Front Genet* **6**, 199 (2015).

12. Bergman, J. E. H. *et al.* Two related Dutch families with a clinically variable presentation of cardioskeletal myopathy caused by a novel S13F mutation in the desmin gene. *Eur J Med Genet* **50**, 355–366 (2007).

13. van Tintelen, J. P. *et al.* Severe cardiac phenotype with right ventricular predominance in a large cohort of patients with a single missense mutation in the DES gene. *Heart Rhythm* **6**, 1574–1583 (2009).

14. Pica, E. C., Kathirvel, P., Pramono, Z. A. D., Lai, P.-S. & Yee, W.-C. Characterization of a novel S13F desmin mutation associated with desmin myopathy and heart block in a Chinese family. *Neuromuscul Disord* **18**, 178–182 (2008).

15. Posey, J. E. *et al.* Molecular diagnostic experience of whole-exome sequencing in adult patients. *Genet Med* **18**, 678–685 (2016).

16. Nicolau, S., Howe, B. M. & Naddaf, E. Novel Desmin Mutation Causing Myofibrillar Myopathy in a Hmong Family. *Front Neurol* **10**, 1375 (2019).

17. Arbustini, E. *et al.* Desmin accumulation restrictive cardiomyopathy and atrioventricular block associated with desmin gene defects. *Eur J Heart Fail* **8**, 477–483 (2006).

18. Vincent, A. E. *et al.* Mitochondrial dysfunction in myofibrillar myopathy. *Neuromuscul Disord* **26**, 691–701 (2016).

19. Walsh, R. *et al.* Reassessment of Mendelian gene pathogenicity using 7,855 cardiomyopathy cases and 60,706 reference samples. *Genet Med* **19**, 192–203 (2017).

20. Lin, Y. *et al.* Applying High-Resolution Variant Classification to Cardiac Arrhythmogenic Gene Testing in a Demographically Diverse Cohort of Sudden Unexplained Deaths. *Circ Cardiovasc Genet* **10**, e001839 (2017).

21. Kubánek, M. *et al.* Desminopathy: Novel Desmin Variants, a New Cardiac Phenotype, and Further Evidence for Secondary Mitochondrial Dysfunction. *J Clin Med* **9**, 937 (2020).

22. VCV000421043.2 - ClinVar - NCBI. https://www.ncbi.nlm.nih.gov/clinvar/variation/421043/.

23. Baker, L. K. *et al.* Nebulin binding impedes mutant desmin filament assembly. *Mol Biol Cell* **24**, 1918–1932 (2013).

24. Yu, M. *et al.* Mutational spectrum of Chinese LGMD patients by targeted next-generation sequencing. *PLoS One* **12**, e0175343 (2017).

25. van Spaendonck-Zwarts, K. Y. *et al.* Desmin-related myopathy. *Clin Genet* **80**, 354–366 (2011).

26. Santori, M. *et al.* Broad-based molecular autopsy: a potential tool to investigate the involvement of subtle cardiac conditions in sudden unexpected death in infancy and early childhood. *Arch Dis Child* **100**, 952–956 (2015).

27. van Lint, F. H. M. *et al.* Large next-generation sequencing gene panels in genetic heart disease: yield of pathogenic variants and variants of unknown significance. *Neth Heart J* **27**, 304–309 (2019).

28. Khan, R. S. *et al.* Genotype and Cardiac Outcomes in Pediatric Dilated Cardiomyopathy. *J Am Heart Assoc* **11**, e022854 (2022).

29. Pugh, T. J. *et al.* The landscape of genetic variation in dilated cardiomyopathy as surveyed by clinical DNA sequencing. *Genet Med* **16**, 601–608 (2014).

30. Miszalski-Jamka, K. *et al.* Novel Genetic Triggers and Genotype-Phenotype Correlations in Patients With Left Ventricular Noncompaction. *Circ Cardiovasc Genet* **10**, e001763 (2017).

31. Norton, N. *et al.* Evaluating pathogenicity of rare variants from dilated cardiomyopathy in the exome era. *Circ Cardiovasc Genet* **5**, 167–174 (2012).

32. Horvat, C. *et al.* A gene-centric strategy for identifying disease-causing rare variants in dilated cardiomyopathy. *Genet Med* **21**, 133–143 (2019).

33. Protonotarios, A. *et al.* The Novel Desmin Variant p.Leu115Ile Is Associated With a Unique Form of Biventricular Arrhythmogenic Cardiomyopathy. *Can J Cardiol* **37**, 857–866 (2021).

34. Klauke, B. *et al.* De novo desmin-mutation N116S is associated with arrhythmogenic right ventricular cardiomyopathy. *Hum Mol Genet* **19**, 4595–4607 (2010).

35. Kant, S., Krusche, C. A., Gaertner, A., Milting, H. & Leube, R. E. Loss of plakoglobin immunoreactivity in intercalated discs in arrhythmogenic right ventricular cardiomyopathy: protein mislocalization versus epitope masking. *Cardiovasc Res* **109**, 260–271 (2016).

36. Maerkens, A. *et al.* Differential proteomic analysis of abnormal intramyoplasmic aggregates in desminopathy. *J Proteomics* **90**, 14–27 (2013).

37. Brodehl, A., Gaertner-Rommel, A. & Milting, H. Molecular insights into cardiomyopathies associated with desmin (DES) mutations. *Biophys Rev* **10**, 983–1006 (2018).

38. VCV000201726.7 - ClinVar - NCBI. https://www.ncbi.nlm.nih.gov/clinvar/variation/201726/.

39. Brodehl, A. *et al.* The novel desmin mutant p.A120D impairs filament formation, prevents intercalated disk localization, and causes sudden cardiac death. *Circ Cardiovasc Genet* **6**, 615–623 (2013).

40. Brodehl, A. *et al.* Restrictive Cardiomyopathy is Caused by a Novel Homozygous Desmin (DES) Mutation p.Y122H Leading to a Severe Filament Assembly Defect. *Genes (Basel)* **10**, 918 (2019).

41. VCV000977180.1 - ClinVar - NCBI. https://www.ncbi.nlm.nih.gov/clinvar/variation/977180/.

42. Haas, J. *et al.* Atlas of the clinical genetics of human dilated cardiomyopathy. *Eur Heart J* **36**, 1123–1135a (2015).

43. Golbus, J. R. *et al.* Targeted analysis of whole genome sequence data to diagnose genetic cardiomyopathy. *Circ Cardiovasc Genet* **7**, 751–759 (2014).

44. VCV000265811.5 - ClinVar - NCBI. https://www.ncbi.nlm.nih.gov/clinvar/variation/265811/.

45. Wilson, K. D. *et al.* A Rapid, High-Quality, Cost-Effective, Comprehensive and Expandable Targeted Next-Generation Sequencing Assay for Inherited Heart Diseases. *Circ Res* **117**, 603–611 (2015).

46. Brodehl, A. *et al.* Functional characterization of the novel DES mutation p.L136P associated with dilated cardiomyopathy reveals a dominant filament assembly defect. *J Mol Cell Cardiol* **91**, 207–214 (2016).

47. Proost, D. *et al.* Targeted Next-Generation Sequencing of 51 Genes Involved in Primary Electrical Disease. *J Mol Diagn* **19**, 445–459 (2017).

48. Ji, J. *et al.* A semiautomated whole-exome sequencing workflow leads to increased diagnostic yield and identification of novel candidate variants. *Cold Spring Harb Mol Case Stud* **5**, a003756 (2019).

49. Campuzano, O. *et al.* Reanalysis and reclassification of rare genetic variants associated with inherited arrhythmogenic syndromes. *EBioMedicine* **54**, 102732 (2020).

50. Gonzalez-Quereda, L. *et al.* Targeted Next-Generation Sequencing in a Large Cohort of Genetically Undiagnosed Patients with Neuromuscular Disorders in Spain. *Genes (Basel)* **11**, 539 (2020).

51. Dal Ferro, M. *et al.* Association between mutation status and left ventricular reverse remodelling in dilated cardiomyopathy. *Heart* **103**, 1704–1710 (2017).

52. Tamiya, R. *et al.* Desmin-related myopathy characterized by non-compaction cardiomyopathy, cardiac conduction defect, and coronary artery dissection. *ESC Heart Fail* **7**, 1338–1343 (2020).

53. Sanchez, O. *et al.* Natural and Undetermined Sudden Death: Value of Post-Mortem Genetic Investigation. *PLoS One* **11**, e0167358 (2016).

54. Goldfarb, L. G., Vicart, P., Goebel, H. H. & Dalakas, M. C. Desmin myopathy. *Brain* **127**, 723–734 (2004).

55. Kostareva, A. *et al.* Desmin A213V substitution represents a rare polymorphism but not a mutation and is more prevalent in patients with heart dilation of various origins. *Acta Myol* **30**, 42–45 (2011).

56. Lorenzon, A. *et al.* Desmin mutations and arrhythmogenic right ventricular cardiomyopathy. *Am J Cardiol* **111**, 400–405 (2013).

57. Bär, H., Strelkov, S. V., Sjöberg, G., Aebi, U. & Herrmann, H. The biology of desmin filaments: how do mutations affect their structure, assembly, and organisation? *J Struct Biol* **148**, 137–152 (2004).

58. Bär, H. *et al.* Severe muscle disease-causing desmin mutations interfere with in vitro filament assembly at distinct stages. *Proc Natl Acad Sci U S A* **102**, 15099–15104 (2005).

59. Bär, H. *et al.* Forced expression of desmin and desmin mutants in cultured cells: impact of myopathic missense mutations in the central coiled-coil domain on network formation. *Exp Cell Res* **312**, 1554–1565 (2006).

60. Bär, H. *et al.* Impact of disease mutations on the desmin filament assembly process. *J Mol Biol* **360**, 1031–1042 (2006).

61. Goudeau, B. *et al.* Variable pathogenic potentials of mutations located in the desmin alpha-helical domain. *Hum Mutat* **27**, 906–913 (2006).

62. Kostareva, A. *et al.* Desmin mutations in a St. Petersburg cohort of cardiomyopathies. *Acta Myol* **25**, 109–115 (2006).

63. Taylor, M. R. G. *et al.* Prevalence of desmin mutations in dilated cardiomyopathy. *Circulation* **115**, 1244–1251 (2007).

64. Goldfarb, L. G. & Dalakas, M. C. Tragedy in a heartbeat: malfunctioning desmin causes skeletal and cardiac muscle disease. *J Clin Invest* **119**, 1806–1813 (2009).

65. Zimmerman, R. S. *et al.* A novel custom resequencing array for dilated cardiomyopathy. *Genet Med* **12**, 268–278 (2010).

66. van Spaendonck-Zwarts, K. Y. *et al.* Recurrent and founder mutations in the Netherlands: the cardiac phenotype of DES founder mutations p.S13F and p.N342D. *Neth Heart J* **20**, 219–228 (2012).

67. Clemen, C. S., Herrmann, H., Strelkov, S. V. & Schröder, R. Desminopathies: pathology and mechanisms. *Acta Neuropathol* **125**, 47–75 (2013).

68. Savarese, M. *et al.* MotorPlex provides accurate variant detection across large muscle genes both in single myopathic patients and in pools of DNA samples. *Acta Neuropathol Commun* **2**, 100 (2014).

69. Beck, T. F. *et al.* FBN1 contributing to familial congenital diaphragmatic hernia. *Am J Med Genet A* **167A**, 831–836 (2015).

70. Monies, D. *et al.* The landscape of genetic diseases in Saudi Arabia based on the first 1000 diagnostic panels and exomes. *Hum Genet* **136**, 921–939 (2017).

71. Harada, H. *et al.* Phenotypic expression of a novel desmin gene mutation: hypertrophic cardiomyopathy followed by systemic myopathy. *J Hum Genet* **63**, 249–254 (2018).

72. Truszkowska, G. T. *et al.* A study in Polish patients with cardiomyopathy emphasizes pathogenicity of phospholamban (PLN) mutations at amino acid position 9 and low penetrance of heterozygous null PLN mutations. *BMC Med Genet* **16**, 21 (2015).

73. Cohen, R. B. *et al.* QT interval dynamics and triggers for QT prolongation immediately following cardiac arrest. *Resuscitation* **162**, 171–179 (2021).

74. Yu, R., Liu, L., Chen, C. & Shen, J.-M. Exome Sequencing Identifies a Novel DES Mutation (R227C) in a Chinese Dilated Cardiomyopathy Family. *Cardiology* **137**, 78–82 (2017).

75. Chen, Y. *et al.* Novel trigenic CACNA1C/DES/MYPN mutations in a family of hypertrophic cardiomyopathy with early repolarization and short QT syndrome. *J Transl Med* **15**, 78 (2017).

76. Vrabie, A. *et al.* The enlarging spectrum of desminopathies: new morphological findings, eastward geographic spread, novel exon 3 desmin mutation. *Acta Neuropathol* **109**, 411–417 (2005).

77. Clemen, C. S. *et al.* How much mutant protein is needed to cause a protein aggregate myopathy in vivo? Lessons from an exceptional desminopathy. *Hum Mutat* **30**, E490-499 (2009).

78. Nallamilli, B. R. R. *et al.* Genetic landscape and novel disease mechanisms from a large LGMD cohort of 4656 patients. *Ann Clin Transl Neurol* **5**, 1574–1587 (2018).

79. Conover, G. M., Henderson, S. N. & Gregorio, C. C. A myopathy-linked desmin mutation perturbs striated muscle actin filament architecture. *Mol Biol Cell* **20**, 834–845 (2009).

80. Strach, K. *et al.* Clinical, genetic, and cardiac magnetic resonance imaging findings in primary desminopathies. *Neuromuscul Disord* **18**, 475–482 (2008).

81. Thomson, K. L. *et al.* Analysis of 51 proposed hypertrophic cardiomyopathy genes from genome sequencing data in sarcomere negative cases has negligible diagnostic yield. *Genet Med* **21**, 1576–1584 (2019).

82. Kedia, N. *et al.* Desmin forms toxic, seeding-competent amyloid aggregates that persist in muscle fibers. *Proc Natl Acad Sci U S A* **116**, 16835–16840 (2019).

83. Tse, H.-F. *et al.* Patient-specific induced-pluripotent stem cells-derived cardiomyocytes recapitulate the pathogenic phenotypes of dilated cardiomyopathy due to a novel DES mutation identified by whole exome sequencing. *Hum Mol Genet* **22**, 1395–1403 (2013).

84. VCV000201704.8 - ClinVar - NCBI. https://www.ncbi.nlm.nih.gov/clinvar/variation/201704/.

85. Ng, D. *et al.* Interpreting secondary cardiac disease variants in an exome cohort. *Circ Cardiovasc Genet* **6**, 337–346 (2013).

86. Singh, S. R., Kadioglu, H., Patel, K., Carrier, L. & Agnetti, G. Is Desmin Propensity to Aggregate Part of its Protective Function? *Cells* **9**, 491 (2020).

87. Andreasen, C. *et al.* New population-based exome data are questioning the pathogenicity of previously cardiomyopathy-associated genetic variants. *Eur J Hum Genet* **21**, 918–928 (2013).

88. Sahlin, E. *et al.* Identification of putative pathogenic single nucleotide variants (SNVs) in genes associated with heart disease in 290 cases of stillbirth. *PLoS One* **14**, e0210017 (2019).

89. Mook, O. R. F. *et al.* Targeted sequence capture and GS-FLX Titanium sequencing of 23 hypertrophic and dilated cardiomyopathy genes: implementation into diagnostics. *J Med Genet* **50**, 614–626 (2013).

90. Goldfarb, L. G. *et al.* Missense mutations in desmin associated with familial cardiac and skeletal myopathy. *Nat Genet* **19**, 402–403 (1998).

91. Fischer, D. *et al.* Distinct muscle imaging patterns in myofibrillar myopathies. *Neurology* **71**, 758–765 (2008).

92. Dalakas, M. C. *et al.* Progressive skeletal myopathy, a phenotypic variant of desmin myopathy associated with desmin mutations. *Neuromuscul Disord* **13**, 252–258 (2003).

93. Dalakas, M. C. *et al.* Desmin myopathy, a skeletal myopathy with cardiomyopathy caused by mutations in the desmin gene. *N Engl J Med* **342**, 770–780 (2000).

94. Otten, E. *et al.* Desmin mutations as a cause of right ventricular heart failure affect the intercalated disks. *Heart Rhythm* **7**, 1058–1064 (2010).

95. Sjöberg, G. *et al.* A missense mutation in the desmin rod domain is associated with autosomal dominant distal myopathy, and exerts a dominant negative effect on filament formation. *Hum Mol Genet* **8**, 2191–2198 (1999).

96. Smolina, N., Bruton, J., Sjoberg, G., Kostareva, A. & Sejersen, T. Aggregate-prone desmin mutations impair mitochondrial calcium uptake in primary myotubes. *Cell Calcium* **56**, 269–275 (2014).

97. Carlsson, L. *et al.* Cytoskeletal derangements in hereditary myopathy with a desmin L345P mutation. *Acta Neuropathol* **104**, 493–504 (2002).

98. Horowitz, S. H. & Schmalbruch, H. Autosomal dominant distal myopathy with desmin storage: a clinicopathologic and electrophysiologic study of a large kinship. *Muscle Nerve* **17**, 151–160 (1994).

99. Fichna, J. P. *et al.* Two desmin gene mutations associated with myofibrillar myopathies in Polish families. *PLoS One* **9**, e115470 (2014).

100. Tobita, T. *et al.* Genetic basis of cardiomyopathy and the genotypes involved in prognosis and left ventricular reverse remodeling. *Sci Rep* **8**, 1998 (2018).

101. Winter, L. *et al.* Mutant desmin substantially perturbs mitochondrial morphology, function and maintenance in skeletal muscle tissue. *Acta Neuropathol* **132**, 453–473 (2016).

102. Clemen, C. S. *et al.* The toxic effect of R350P mutant desmin in striated muscle of man and mouse. *Acta Neuropathol* **129**, 297–315 (2015).

103. Bär, H. *et al.* Pathogenic effects of a novel heterozygous R350P desmin mutation on the assembly of desmin intermediate filaments in vivo and in vitro. *Hum Mol Genet* **14**, 1251–1260 (2005).

104. Levin, J. *et al.* Divergent molecular effects of desmin mutations on protein assembly in myofibrillar myopathy. *J Neuropathol Exp Neurol* **69**, 415–424 (2010).

105. Walter, M. C. *et al.* Scapuloperoneal syndrome type Kaeser and a wide phenotypic spectrum of adult-onset, dominant myopathies are associated with the desmin mutation R350P. *Brain* **130**, 1485–1496 (2007).

106. Bonakdar, N. *et al.* Biomechanical characterization of a desminopathy in primary human myoblasts. *Biochem Biophys Res Commun* **419**, 703–707 (2012).

107. Wu, L., Brady, L., Shoffner, J. & Tarnopolsky, M. A. Next-Generation Sequencing to Diagnose Muscular Dystrophy, Rhabdomyolysis, and HyperCKemia. *Can J Neurol Sci* **45**, 262–268 (2018).

108. Shanks, G. W. *et al.* Importance of Variant Interpretation in Whole-Exome Molecular Autopsy: Population-Based Case Series. *Circulation* **137**, 2705–2715 (2018).

109. VCV000947073.5 - ClinVar - NCBI. https://www.ncbi.nlm.nih.gov/clinvar/variation/947073/.

110. Fidziańska, A. *et al.* A novel desmin R355P mutation causes cardiac and skeletal myopathy. *Neuromuscul Disord* **15**, 525–531 (2005).

111. Fischer, D. *et al.* Different early pathogenesis in myotilinopathy compared to primary desminopathy. *Neuromuscul Disord* **16**, 361–367 (2006).

112. Chourbagi, O. *et al.* Desmin mutations in the terminal consensus motif prevent synemin-desmin heteropolymer filament assembly. *Exp Cell Res* **317**, 886–897 (2011).

113. Dagvadorj, A. *et al.* Respiratory insufficiency in desminopathy patients caused by introduction of proline residues in desmin c-terminal alpha-helical segment. *Muscle Nerve* **27**, 669–675 (2003).

114. Kreplak, L. & Bär, H. Severe myopathy mutations modify the nanomechanics of desmin intermediate filaments. *J Mol Biol* **385**, 1043–1051 (2009).

115. Symbas, P. N. & Gott, J. P. Delayed sequelae of thoracic trauma. *Surg Clin North Am* **69**, 135–142 (1989).

116. Olivé, M. *et al.* Phenotypic patterns of desminopathy associated with three novel mutations in the desmin gene. *Neuromuscul Disord* **17**, 443–450 (2007).

117. Arias, M. *et al.* Distinct phenotypic features and gender-specific disease manifestations in a Spanish family with desmin L370P mutation. *Neuromuscul Disord* **16**, 498–503 (2006).

118. Hnia, K. *et al.* Myotubularin controls desmin intermediate filament architecture and mitochondrial dynamics in human and mouse skeletal muscle. *J Clin Invest* **121**, 70–85 (2011).

119. Olivé, M. *et al.* Clinical and myopathological evaluation of early- and late-onset subtypes of myofibrillar myopathy. *Neuromuscul Disord* **21**, 533–542 (2011).

120. Smolina, N. *et al.* Desmin mutations result in mitochondrial dysfunction regardless of their aggregation properties. *Biochim Biophys Acta Mol Basis Dis* **1866**, 165745 (2020).

121. VCV000522692.4 - ClinVar - NCBI. https://www.ncbi.nlm.nih.gov/clinvar/variation/522692/.

122. Sugawara, M. *et al.* A novel de novo mutation in the desmin gene causes desmin myopathy with toxic aggregates. *Neurology* **55**, 986–990 (2000).

123. Zhao, Y. *et al.* Targeted next-generation sequencing of candidate genes reveals novel mutations in patients with dilated cardiomyopathy. *Int J Mol Med* **36**, 1479–1486 (2015).

124. Goudeau, B. *et al.* Structural and functional analysis of a new desmin variant causing desmin-related myopathy. *Hum Mutat* **18**, 388–396 (2001).

125. van Waning, J. I. *et al.* Genetics, Clinical Features, and Long-Term Outcome of Noncompaction Cardiomyopathy. *J Am Coll Cardiol* **71**, 711–722 (2018).

126. Jiménez-Jáimez, J. *et al.* Clinical and Genetic Diagnosis of Nonischemic Sudden Cardiac Death. *Rev Esp Cardiol (Engl Ed)* **70**, 808–816 (2017).

127. Bermúdez-Jiménez, F. J. *et al.* Novel Desmin Mutation p.Glu401Asp Impairs Filament Formation, Disrupts Cell Membrane Integrity, and Causes Severe Arrhythmogenic Left Ventricular Cardiomyopathy/Dysplasia. *Circulation* **137**, 1595–1610 (2018).

128. VCV001501730.3 - ClinVar - NCBI. https://www.ncbi.nlm.nih.gov/clinvar/variation/1501730/.

129. Punetha, J. *et al.* Targeted Re-Sequencing Emulsion PCR Panel for Myopathies: Results in 94 Cases. *J Neuromuscul Dis* **3**, 209–225 (2016).

130. Dagvadorj, A. *et al.* A series of West European patients with severe cardiac and skeletal myopathy associated with a de novo R406W mutation in desmin. *J Neurol* **251**, 143–149 (2004).

131. Olivé, M. *et al.* Desmin-related myopathy: clinical, electrophysiological, radiological, neuropathological and genetic studies. *J Neurol Sci* **219**, 125–137 (2004).

132. Joanne, P. *et al.* Viral-mediated expression of desmin mutants to create mouse models of myofibrillar myopathy. *Skelet Muscle* **3**, 4 (2013).

133. Herrmann, H. *et al.* Dual Functional States of R406W-Desmin Assembly Complexes Cause Cardiomyopathy With Severe Intercalated Disc Derangement in Humans and in Knock-In Mice. *Circulation* **142**, 2155–2171 (2020).

134. VCV002109936.1 - ClinVar - NCBI. https://www.ncbi.nlm.nih.gov/clinvar/variation/2109936/.

135. Pruszczyk, P. *et al.* Restrictive cardiomyopathy with atrioventricular conduction block resulting from a desmin mutation. *Int J Cardiol* **117**, 244–253 (2007).

136. Bär, H. *et al.* Conspicuous involvement of desmin tail mutations in diverse cardiac and skeletal myopathies. *Hum Mutat* **28**, 374–386 (2007).

137. Charrier, E. E. *et al.* Desmin Mutation in the C-Terminal Domain Impairs Traction Force Generation in Myoblasts. *Biophys J* **110**, 470–480 (2016).

138. Goldfarb, L. G., Olivé, M., Vicart, P. & Goebel, H. H. Intermediate filament diseases: desminopathy. *Adv Exp Med Biol* **642**, 131–164 (2008).

139. Alimohamed, M. Z. *et al.* Diagnostic yield of targeted next generation sequencing in 2002 Dutch cardiomyopathy patients. *Int J Cardiol* **332**, 99–104 (2021).

140. Hedberg, C., Melberg, A., Kuhl, A., Jenne, D. & Oldfors, A. Autosomal dominant myofibrillar myopathy with arrhythmogenic right ventricular cardiomyopathy 7 is caused by a DES mutation. *Eur J Hum Genet* **20**, 984–985 (2012).

141. Brodehl, A. *et al.* Functional characterization of desmin mutant p.P419S. *Eur J Hum Genet* **21**, 589–590 (2013).

142. Ripoll-Vera, T. *et al.* Phenotypic Patterns of Cardiomyopathy Caused by Mutations in the Desmin Gene. A Clinical and Genetic Study in Two Inherited Heart Disease Units. *Rev Esp Cardiol (Engl Ed)* **68**, 1027–1029 (2015).

143. JurcuŢ, R. O. *et al.* Discovery of a new mutation in the desmin gene in a young patient with cardiomyopathy and muscular weakness. *Rom J Morphol Embryol* **58**, 225–230 (2017).

144. Claeys, K. G. *et al.* Electron microscopy in myofibrillar myopathies reveals clues to the mutated gene. *Neuromuscul Disord* **18**, 656–666 (2008).

145. Torres, M. P., Dewhurst, H. & Sundararaman, N. Proteome-wide Structural Analysis of PTM Hotspots Reveals Regulatory Elements Predicted to Impact Biological Function and Disease. *Mol Cell Proteomics* **15**, 3513–3528 (2016).

146. Li, D. *et al.* Desmin mutation responsible for idiopathic dilated cardiomyopathy. *Circulation* **100**, 461–464 (1999).

147. Li, M. & Dalakas, M. C. Abnormal desmin protein in myofibrillar myopathies caused by desmin gene mutations. *Ann Neurol* **49**, 532–536 (2001).

148. Miyamoto, Y. *et al.* Frequency and clinical characteristics of dilated cardiomyopathy caused by desmin gene mutation in a Japanese population. *Eur Heart J* **22**, 2284–2289 (2001).

149. S, S. *et al.* αB-crystallin is a sensor for assembly intermediates and for the subunit topology of desmin intermediate filaments. *Cell stress & chaperones* **22**, (2017).

150. Stępień-Wojno, M. *et al.* Sudden cardiac arrest in patients without overt heart disease: a limited value of next generation sequencing. *Pol Arch Intern Med* **128**, 721–730 (2018).

151. Hernandez, D. A. *et al.* Nebulette is a powerful cytolinker organizing desmin and actin in mouse hearts. *Mol Biol Cell* **27**, 3869–3882 (2016).

152. Dias, R. & Aguiar, T. C. DES c.1360C>T: A Rare Desmin Variant Causing Early Distal Myopathy and Cardiomyopathy. *Cureus* **15**, e36368 (2023).

153. Bär, H. *et al.* Mutations in desmin’s carboxy-terminal ‘tail’ domain severely modify filament and network mechanics. *J Mol Biol* **397**, 1188–1198 (2010).

154. Claeys, K. G. *et al.* Differential involvement of sarcomeric proteins in myofibrillar myopathies: a morphological and immunohistochemical study. *Acta Neuropathol* **117**, 293–307 (2009).

155. Ackerman, J. P. *et al.* The Promise and Peril of Precision Medicine: Phenotyping Still Matters Most. *Mayo Clin Proc* S0025-6196(16)30463–3 (2016) doi:10.1016/j.mayocp.2016.08.008.

156. Cerino, M. *et al.* Genetic Characterization of a French Cohort of GNE-mutation negative inclusion body myopathy patients with exome sequencing. *Muscle Nerve* **56**, 993–997 (2017).

157. Gearhart, A. S. & Batra, A. S. Isolated left bundle branch block progressing to complete heart block and asystole: A novel presentation of a desmin mutation. *HeartRhythm Case Rep* **4**, 184–186 (2018).

158. Oomen, A. W. G. J. *et al.* Rare desmin variant causing penetrant life-threatening arrhythmic cardiomyopathy. *HeartRhythm Case Rep* **4**, 318–323 (2018).

159. Parrott, A. *et al.* Investigation of de novo variation in pediatric cardiomyopathy. *Am J Med Genet C Semin Med Genet* **184**, 116–123 (2020).

160. Weihl, C. C. *et al.* Targeted sequencing and identification of genetic variants in sporadic inclusion body myositis. *Neuromuscul Disord* **25**, 289–296 (2015).

161. Nouhravesh, N. *et al.* Analyses of more than 60,000 exomes questions the role of numerous genes previously associated with dilated cardiomyopathy. *Mol Genet Genomic Med* **4**, 617–623 (2016).

162. Muntoni, F. *et al.* Disease severity in dominant Emery Dreifuss is increased by mutations in both emerin and desmin proteins. *Brain* **129**, 1260–1268 (2006).
